# Aggregation and morphology control enables multiple cases of high-efficiency polymer solar cells

**DOI:** 10.1038/ncomms6293

**Published:** 2014-11-10

**Authors:** Yuhang Liu, Jingbo Zhao, Zhengke Li, Cheng Mu, Wei Ma, Huawei Hu, Kui Jiang, Haoran Lin, Harald Ade, He Yan

**Affiliations:** 1 Department of Chemistry, The Hong Kong University of Science and Technology, Clear Water Bay, Kowloon, Hong Kong; 2 Department of Physics and ORaCEL, North Carolina State University, Raleigh, North Carolina 27695, USA; 3 HKUST-Shenzhen Research Institute, No. 9 Yuexing 1st RD, Hi-tech Park, Nanshan, Shenzhen 518057, China

## Abstract

Although the field of polymer solar cell has seen much progress in device performance in the past few years, several limitations are holding back its further development. For instance, current high-efficiency (>9.0%) cells are restricted to material combinations that are based on limited donor polymers and only one specific fullerene acceptor. Here we report the achievement of high-performance (efficiencies up to 10.8%, fill factors up to 77%) thick-film polymer solar cells for multiple polymer:fullerene combinations via the formation of a near-ideal polymer:fullerene morphology that contains highly crystalline yet reasonably small polymer domains. This morphology is controlled by the temperature-dependent aggregation behaviour of the donor polymers and is insensitive to the choice of fullerenes. The uncovered aggregation and design rules yield three high-efficiency (>10%) donor polymers and will allow further synthetic advances and matching of both the polymer and fullerene materials, potentially leading to significantly improved performance and increased design flexibility.

## Abstract (web summary)

Polymer solar cells promise a cost-effective way to harness solar energy, but cell performance is held back by limited choices of suitable materials. Here, Liu *et al.* demonstrate record cell efficiencies for multiple material combinations via a new approach of aggregation and morphology control.

## Graphical abstract

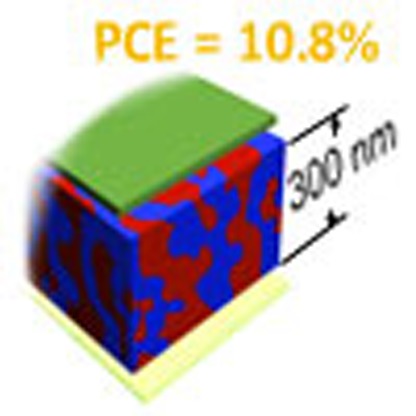

Conventional inorganic solar cells can achieve high efficiencies but are produced through complicated, costly processes. The desirability of lower costs is driving the development of several third-generation solar technologies. Among these, polymer solar cell (PSC)^1^,^2^,^3^,^4^,^5^,^6^ technology is an excellent example of low-cost production because PSCs can be produced using extremely high-throughput roll-to-roll printing methods similar to those used to print newspapers^7^. PSCs also offer several other advantages: vacuum processing and high-temperature sintering are not needed, and no toxic materials are used in the end product. Most importantly, a tandem cell architecture^6^,^8^,^9^,^10^ can be easily implemented with PSCs and has proven to improve PSC efficiency by ~40–50% (refs 6, 8). As PSCs are two-component, donor–acceptor material systems, it is generally important to control the morphology of the donor:acceptor blends and to find an optimal materials combination with excellent optical and electronic properties. In the last few years, record-efficiency PSCs were achieved with only three donor polymers (which all belong to a specific polymer family based on fluorinated thieno[3,4-*b*]thiophene, for example, PTB7) that are, furthermore, constrained to be used with a specific fullerene, PC_71_BM, to achieve their best performance^11^,^12^,^13^. In general, the morphologies^14^,^15^,^16^ and thus performance of state-of-the-art donor polymers (for example, PTB7 (refs 11, 17) and PBDT-DTNT^18^) are sensitive to the choice of fullerene and replacing PC_71_BM with another C_60_-based or non-PCBM fullerene decreases PSC efficiency to 6-7% (refs 11, 18, 19, 20) The dominant role of PC_71_BM places serious constraints on PSC material development, because the properties of the polymers must be precisely matched with fixed targets set by PC_71_BM. As tandem PSCs require two sets of perfectly matching polymer/fullerene materials, the constraint on their development is compounded. It has thus been pointed out that it is crucial to have the flexibility of being able to use different fullerenes and more generally to remove material constraints to achieve tandem PSCs with 15–20% efficiency envisioned by Brebac and colleagues^9^,^10^,^21^. The development of polymer:fullerene material systems that are morphologically insensitive to fullerene choice will remove these material constraints, and greatly accelerate material development for single-junction and tandem PSCs^10^,^22^.

Another important fundamental issue for the PSC field is how to control the morphology of polymer:fullerene blends to achieve the best PSC performance. There is likely more than one near-optimum PSC morphology. The famous PTB7 family donor polymers enabled one type of the near-optimum PSC morphology, as high external quantum efficiencies (EQEs~80%) have been reported for PTB7-based cells^11^. However, the PTB7-based PSC materials and devices have certain limitations. Besides the sensitivity of the choice of fullerenes, another important limitation for PTB7 family polymers is that they cannot perform well when relatively thick active layers (~300 nm) are used in the PSC device. Thick-film PSCs are important for the industrial application of PSCs, and thick films should also further increase the absorption strength of the solar cell and thus cell efficiency. The reason why PTB7 does not perform well in thick-film PSCs is partially owing to the relatively low hole transport ability (space charge limited current (SCLC) mobility ~6 × 10^−4^ cm^2^ V^−1^ s^−1^; ref. 17) related to the low crystallinity of the PTB7 polymer. There has been also evidence that high purity of the polymer domain may be an important factor to achieve efficient thick-film PSCs^14^,^23^,^24^. The PTB7-based materials systems are characterized by relatively impure polymer domains^25^, which could be a reason why these polymers do not perform well in thick-film PSCs. Clearly, there is a need for new materials systems that explore a different ‘near-optimum’ PSC morphology in order to achieve thick-film PSCs that have comparable or higher efficiencies than state-of-the-art PTB7 materials systems.

In the following, we report the achievement of high-performance (efficiencies up to 10.8% and fill factors (FFs) up to 77%) thick-film PSCs based on three different donor polymers and 10 polymer:fullerene combinations, all of which exhibit efficiencies higher than the previous state of the art. In contrast to state-of-the-art PTB7-based materials systems, the high PSC performances in this report are achieved via the formation of an ‘optimum PSC morphology’ that contains highly crystalline, sufficiently pure, yet reasonably small polymer domains. The high polymer crystallinity and thus excellent hole transport ability, combined with sufficiently pure polymer domains, are the main reasons why the PSCs exhibit high FFs and efficiency even when the active layer is 300 nm thick. Importantly, this *ipso facto* near-perfect morphology is controlled by the temperature-dependent aggregation behaviour of the donor polymers during casting and is insensitive to the choice of fullerenes. Taking advantage of the robust polymer:fullerene morphology enabled by the three donor polymers, many non-traditional fullerenes are also used. Traditional PCBMs, the most dominant fullerenes in PSCs, are out-performed by several other non-traditional fullerenes, clearly indicating the benefits of exploring different fullerenes and the robust morphology formation. Comparative studies on several structurally similar polymers reveal that the 2-octyldodecyl (2OD) alkyl chains sitting on quaterthiophene is the key structural feature that causes the polymers’ highly temperature-dependent aggregation behaviour that allows for the processing of the polymer solutions at elevated temperature, and, more importantly, controlled aggregation and strong crystallization of the polymer during the film cooling and drying process. The branching position and size of the branched alkyl chains are critically important in enabling an optimal aggregation behaviour. With our approach, PSC production is no longer constrained by the use of a single fullerene or by a very thin active layer. Our aggregation and morphology control approach and polymer design rules can be applied to multiple polymer:fullerene materials systems and will allow the PSC community to explore many more polymers and fullerene materials and to optimize their combinations (energy offsets, bandgap and so on) under a well-controlled morphological landscape, which would greatly accelerate the materials and process development towards improved PSCs.

## Results

### PSC device performance

**Figure 1:**
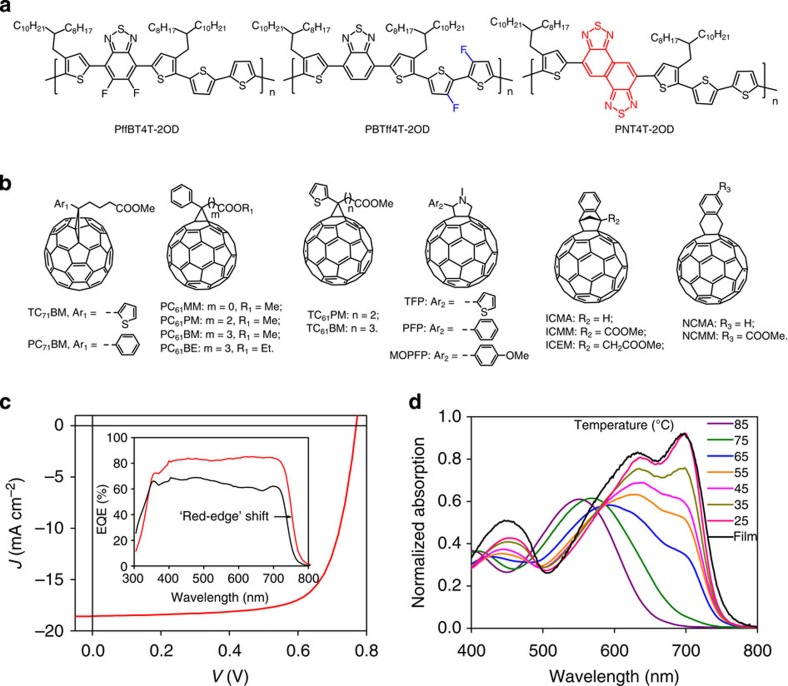
Chemical structures and optical and photovoltaic properties. (**a**,**b**) Chemical structures of donor polymers and fullerenes; (**c**) *J–V* curve of a PffBT4T-2OD:PC_71_BM cell under AM1.5G illumination with an irradiation intensity of 100 mW cm^−2^ (one Sun). Inset: representative EQE spectra of PSCs with a thick (300 nm) and thin (150 nm) active layer. The arrow indicates the shift of the ‘low energy edge’ of the PSCs. (**d**) Ultraviolet–visible (UV-Vis) absorption spectra of a PffBT4T-2OD film and a PffBT4T-2OD solution (0.02 mg ml^−1^ in DCB) at temperatures as indicated.

Among the three donor polymers, we developed that achieved power conversion efficiency>10%, we first focus on poly[(5,6-difluoro-2,1,3-benzothiadiazol-4,7-diyl)-*alt*-(3,3′′′-di(2-octyldodecyl)-2,2′;5′,2′′;5′′,2′′′-quaterthiophen-5,5′′′-diyl)], PffBT4T-2OD (Fig. 1a). PffBT4T-2OD is a material that enables six cases of high-efficiency (9.6–10.8%), high FF (73–77%) and thick-film (250–300 nm) PSCs (Table 1) when combined with traditional PCBM and many non-traditional fullerenes (Fig. 1b). A typical *J*–*V* plot of a PffBT4T-2OD:fullerene PSC is shown in Fig. 1c, with EQE spectra shown in the inset. The benefits of thick-film PSCs are obvious. The thick cell exhibits 10–20% higher EQE values, and the effective absorption bandwidth of a thick PSC can be increased as the result of a ~20 nm red-shift of the ‘leading, low energy edge’ of a PSC’s EQE spectrum. Combined, these account for a ~30% increase in short circuit current (*J*_SC_). Taking advantage of PffBT4T-2OD’s excellent aggregation properties (as delineated further below), we synthesized more than a dozen known or new fullerene derivatives (Fig. 1b) to find the best acceptor match for PffBT4T-2OD. All of these fullerenes form similar morphologies with PffBT4T-2OD and can produce PSCs with high efficiencies in the range of 8.6–10.8% (Table 1 and Supplementary Table 1). The best efficiency (10.4%) in the C_60_ family was achieved by PC_61_PM (Fig. 1b), and the most commonly used C_60_-based fullerene, PC_61_BM, is not the best match for PffBT4T-2OD.

### Polymer crystallinity and hole mobility

**Figure 2:**
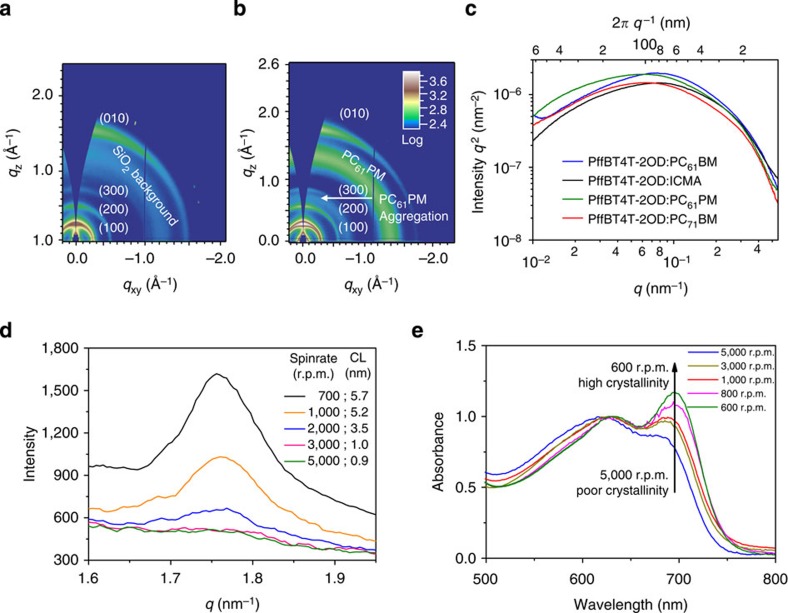
Morphological characterization data. (**a**) Two-dimensional (2D) GIWAXS pattern of a pure PffBT4T-2OD film. (**b**) 2D GIWAXS pattern of a PffBT4T-2OD:PC_61_PM. (**c**) R-SoXS profiles in log scale for four samples of PffBT4T-2OD:fullerene blends. Blue line: PC_61_BM; red line: PC_71_BM; black line: ICMA; green line: PC_61_PM. (**d**) (010) diffraction peak (obtained from XRD) of PffBT4T-2OD pure films spun at different rates, the inset indicates the (010) coherence length (CL) of the films. (**e**) UV-Vis absorption spectra of PffBT4T-2OD:PC_61_PM blend films obtained with different spin rates and substrate temperatures. Blue line, 5,000 r.p.m./110 °C; dark green line, 3,000 r.p.m./100 °C; red line, 1,000 r.p.m./90 °C; pink line, 800 r.p.m./80 °C; emerald line, 600 r.p.m./70 °C. All spectra are normalized based on the intensity of their 0-1 transition peak (at ~640 nm) to highlight the change of the intensity of 0-0 transition peaks.

Grazing incident wide-angle X-ray diffraction (GIWAXS)^26^ reveals the molecular packing and orientational texture of pure PffBT4T-2OD and PffBT4T-2OD:fullerene blend films. Both exhibit a high degree of molecular order, as evidenced by strong lamellar (100), (200) and even (300) reflection peaks and, more importantly, a large (010) coherence length (GIWAXS 2D patterns shown in Fig. 2a,b and Supplementary Fig. 1). The (010) coherence length (that is, extent of ordering) of PffBT4T-2OD:PC_61_PM blend films was calculated using Scherrer analysis^27^ to be ~8.5 nm, which corresponds to ~24 *π*-stacked copolymers. In contrast, the (010) coherence length of PTB7:PC_61_BM, for example, is only ~2 nm (ref. 16). Owing to the high crystallinity and preferential face-on orientation of polymer domains, relatively high SCLC hole mobility of 1.5–3.0 × 10^−2^ cm^2^ V^−1^ s^−1^ were obtained for various PffBT4T-2OD:fullerene blend films in a hole-only diode device configuration (Supplementary Fig. 2). The importance of mobility for good FF was recently illustrated^23^.

### Polymer:fullerene domain size and average domain purity

**Figure 3:**
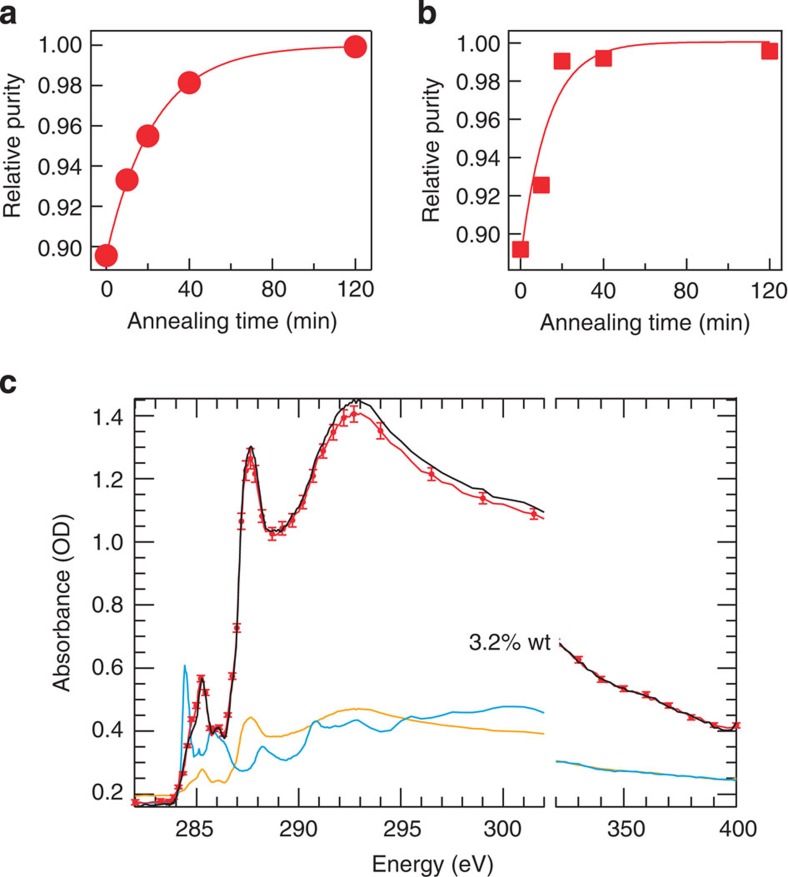
Domain purity data and analysis. (**a**) R-SoXS results revealing relative purity of PffBT4T-2OD:ICMA and (**b**) PffBT4T-2OD:PC_71_BM blends annealed at 130 °C. (**c**) Following methodology developed by Collins *et al.*,^40^ residual ICMA in PffBT4T-2OD is only 3.2% after a PffBT4T-2OD:ICMA blend has been annealed extensively until all excess ICMA has agglomerated into macro-phase domains or crystals. Red lines/symbols are near edge X-ray absorption fine structure data/uncertainty and black lines are fits. Yellow and blue lines are PffBT4T-2OD and fullerene reference spectra used in the fit. Error bars represent the average of three different spots on the sample. A value of 3.2% indicates that there are 3.2% (w.t) fullerene in the average polymer domains that includes polymer crystals and the mixed, amorphous phase.

In addition, resonant soft X-ray scattering^14^,^15^,^25^,^28^,^29^,^30^ (R-SoXS; Fig. 2c) and atomic force microscopy (AFM; Supplementary Fig. 3) analysis reveals that the various PffBT4T-2OD:fullerene films all exhibit multi-length scale morphologies with reasonably small median domain sizes of ~30–40 nm, which is similar to previous cases of high-performance polymers^16^,^25^. R-SoXS can also reveal the average composition variations, which are indicative of the average purity of the polymer and fullerene regions as well as a possible third phase of polymer-rich domains^26^,^31^. An annealing sequence on PffBT4T-2OD:fullerene blends revealed that the non-annealed devices presented here exhibited almost 90% average purity compared with the asymptotic limit (Fig. 3a,b), which corresponds to an unusually low residual concentration of 3.2% fullerene averaged over all PffBT4T-2OD domains in the film as measured by X-ray microscopy (Fig. 3c)^25^,^32^,^33^. In general, PSCs with significantly impure polymer phases exhibit detrimental bimolecular charge recombination when the polymer film is too thick, whereas pure phases can help to minimize recombination^23^. These morphological data show that PffBT4T-2OD can form a polymer:fullerene morphology containing highly crystalline and sufficiently pure yet reasonably small polymer domains. Note that PTB7-type polymers have been the best donor polymer in PSCs for the past few years. By its very nature of high performance in thin films, PTB7 can form a ‘near-optimum’ PSC morphology characterized by relatively low molecular ordering, relatively low hole mobilities and impure polymer domains^25^. PffBT4T-2OD exhibits high molecular ordering (‘crystallinity’), high hole mobilities and purer polymer domains, which appears to be a different *ipso facto* ‘near-optimum’ PSC morphology. Although PTB7 enabled great thin-film PSC performance, PffBT4T-2OD offers high performance even in thick-film PSCs owing to the high mobility of the highly ordered and sufficiently pure polymer domains it forms.

### Morphology control via temperature-dependent aggregation

We attribute PffBT4T-2OD’s excellent performance and robust morphology to its significant temperature-dependent aggregation behaviour that can be exploited during device fabrication. The UV-Vis absorption spectra exhibit a marked red-shift when a low concentration PffBT4T-2OD solution in 1,2-dichlorobenzene (DCB) is lowered from 85 to 25 °C (Fig. 1d). At elevated temperature, PffBT4T-2OD is well dissolved and disaggregated. At progressively lower temperatures, a strong 0-0 transition peak at ~700 nm emerges with significant strength at 25 °C, indicating strong aggregation of the polymer chains in solution at that temperature. Note that the absorption spectrum of the 25 °C solution of PffBT4T-2OD is almost identical to that of the optimized PffBT4T-2OD solid film (Fig. 1d), which is observed to be highly crystalline by GIWAXS. Consequently, devices are always cast from warm solutions (60–80 °C) of PffBT4T-2OD, which then aggregates during the cooling and film-forming process.

To understand details of PffBT4T-2OD’s aggregation behaviour during the film-forming process, the critical *π*–*π* molecular ordering ((010) coherence length and intensity of the (010) peak) is determined with X-ray diffraction (XRD) for a series of PffBT4T-2OD films spun at different rates. As shown in Fig. 2d and Supplementary Table 2, the *π*–*π* ordering decreases markedly with increasing spin rates. As PffBT4T-2OD exhibits a strong yet progressively evolving aggregation property, the extent of PffBT4T-2OD’s aggregation depends upon temperature and concentration changes, the film drying time and the kinetics of aggregation. During a slow spin process (for example, 700 r.p.m.), it takes a relatively long time for the solution and film to dry, during which the temperature of the substrate and the wet film also decreases significantly. When using an ultra-fast rate (for example, 5,000 r.p.m.), however, the solvent evaporates more quickly, which results in kinetically quenched, poorly ordered films, whereas slow spin rates provides PffBT4T-2OD sufficient time to aggregate and to form crystalline polymer domains with large coherence lengths. Importantly, studies for PffBT4T-2OD pure films and PffBT4T-2OD blend films with two different fullerenes yield similar trends (Supplementary Table 2 and Supplementary Fig. 4), demonstrating that the aggregation of PffBT4T-2OD is insensitive to the presence of fullerenes. Not surprisingly, high substrate temperatures were found to have a similar effect to fast spin rates. PffBT4T-2OD:fullerene films prepared with fast spin rates/high substrate temperatures show a decrease in the 0-0 transition peaks and a pronounced shift in the 0-0 transition energy in their UV-Vis absorption spectra, indicative of significant disorder (Fig. 2e). The corresponding hole-only and PSC devices fabricated using high spin rates and high substrate temperature also exhibit markedly decreased hole mobilities (3.1 × 10^−3^ cm^2^ V^−1^ s^−1^; Supplementary Table 3) and PSC efficiencies (3.6%; Supplementary Table 4). These morphological, spectroscopic and electric data demonstrate that PffBT4T-2OD’s morphology is mainly controlled by the progress of its aggregation during the film-casting process until the film is dry, which locks-in the length scale of the morphology. PffBT4T-2OD’s strong yet well-controllable aggregation property allows for convenient optimization of processing conditions that led to a near-ideal polymer:fullerene morphology that is insensitive to the choices of fullerene. This approach of controlling the extent of polymer aggregation during a warm solution casting process is different from the common processing protocol of PTB7 family polymers that are typically processed at room temperature.

## Discussion

**Figure 4:**
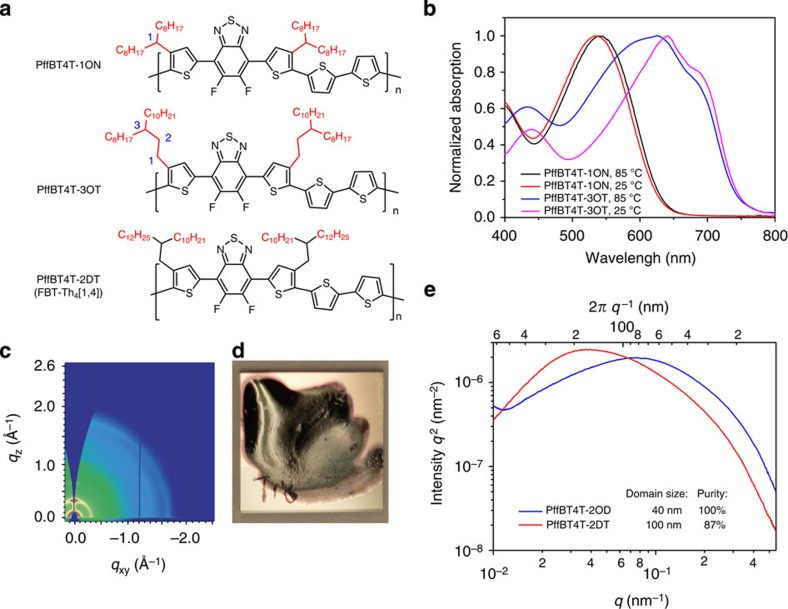
Comparative studies on structurally similar polymers. (**a**) Chemical structures of PffBT4T-1ON, PffBT4T-3OT and PffBT4T-2DT. (**b**) Normalized UV-Vis absorption spectra of PffBT4T-1ON and PffBT4T-3OT in a 85 °C DCB solution and in a DCB solution at room temperature as indicated on the plot. (**c**) Two-dimensional GIWAXS pattern of a pure PffBT4T-1ON film. (**d**) Uneven film formed by the PffBT4T-3OT solution forming a gel before being able to be spun. (**e**) R-SoXS profiles in log scale for PffBT4T-2OD and PffBT4T-2DT, the inset indicates the domain size and relative purity.

The key structural feature of PffBT4T-2OD that enables its pronounced, yet gradual temperature-dependent aggregation (Fig. 1d) is the second-position branched alkyl chains (2OD) on a quaterthiophene (4T-2OD). To elucidate this aspect, we contrast PffBT4T-2OD with two structurally very similar polymers. These two polymers have the same backbone but their alkyl chains are branched at the first or third side-chain carbon atom (for ease of comparison, these two polymers are named as PffBT4T-1ON and PffBT4T-3OT; Fig. 4a). In contrast to PffBT4T-2OD, PffBT4T-1ON is disaggregated at 85 °C, and more importantly, also disaggregated at 25 °C (Fig. 4b). As a result, PffBT4T-1ON cannot aggregate easily during the film-forming process, leading to films with poor crystallinity (GIWAXS pattern shown in Fig. 4c) and thus PSC devices of only ~0.6% efficiency. PffBT4T-3OT exhibits the other extreme, showing excessive aggregation at both 25 and 85 °C. During our attempt to process PffBT4T-3OT, the PffBT4T-3OT solution quickly becomes a gel (Fig. 4d) even before the start of spin casting. These comparisons indicate that PffBT4T-1ON’s alkyl chains cause too much steric hindrance, which results in poor aggregation and crystallinity. PffBT4T-3OT’s alkyl chains provide too little steric hindrance that makes aggregation of PffBT4T-3OT too strong even at 85 °C and makes it difficult to process. PffBT4T-2OD’s second-position branched alkyl chains offer an optimal tradeoff that allows for controllable aggregation of PffBT4T-2OD during the film-forming process. More discussions on the impact of alkyl chain branching positions are provided in Supplementary Note 1.

Several structurally similar donor polymers containing quaterthiophene substituted with second-position branched alkyl chains were reported in the literature^34^,^35^,^36^,^37^, including a recent report in which a polymer with longer alkyl chains, FBT-Th_4_(1,4) (named as PffBT4T-2DT for simplicity in this paper, structure shown in Fig. 4a), achieved 7.64% efficiency^34^. The difference of PffBT4T-2OD and PffBT4T-2DT devices can be understood from the following results. R-SoXS (Fig. 4e) and prior AFM studies^34^ show that the average domain size of PffBT4T-2DT:fullerene films is too large (~100 nm). The R-SoXS data furthermore show that the average purity of the polymer/polymer-rich domains in an PffBT4T-2DT:fullerene film is ~87% of that in PffBT4T-2OD:fullerene film. Lower purity of average polymer/polymer-rich domains can result in significant recombination for thick-film PSC devices and thus lower performance^14^,^23^,^24^. Regarding molecular ordering, the two-dimensional GIWAXS mapping of PffBT4T-2DT:PC_71_BM films^34^ shows that PffBT4T-2DT:fullerene films exhibit weak laminar packing peaks, which are significantly weaker than those of PffBT4T-2OD:fullerene films. The smaller degree of laminar packing of PffBT4T-2DT is consistent with the lower average purity of PffBT4T-2DT polymer domains compared with that of PffBT4T-2OD’s, as more fullerene is expelled by the crystalline polymer domains of PffBT4T-2OD. Lastly, the absorption coefficient of PffBT4T-2DT is lower than that of PffBT4T-2OD owing to longer alkyl chains that does not contribute to light absorption (Supplementary Fig. 5). These studies show that the branching position and the size of the alkyl chains are critically important in obtaining the optimal aggregation properties of PffBT4T-2OD. Insufficient aggregation (for example, PffBT4T-1ON) and unnecessarily long alkyl chains (for example, PffBT4T-2DT) resulted in low crystallinity and/or impure polymer domains. Excessive aggregation (for example, PffBT4T-3OT) makes the processing and aggregation difficult to control. Similar to recent observations^32^,^36^,^38^, the molecular weight of PffBT4T-2OD has a significant impact on its aggregation property and performance. Lower molecular weight (*M*_n_=16.6 kDa, *M*_w_=29.5 kDa) batches of PffBT4T-2OD exhibit weaker aggregation and thus lower efficiency (7.7%) than the high molecular weight (*M*_n_=47.5 kDa, *M*_w_=93.7 kDa) PffBT4T-2OD batches (Supplementary Table 5 and Supplementary Fig. 6). The impacts of polymer molecular weight on PSC performances are discussed in details in the Supplementary Note 2.

Following the rationale described above, we synthesized two other polymers (poly[(2,1,3-benzothiadiazol-4,7-diyl)-*alt*-(4′,3′′-difluoro-3,3′′′-di(2-octyldodecyl)-2,2′;5′,2′′;5′′,2′′′-quaterthiophen-5,5′′′-diyl)] (PBTff4T-2OD) and poly[(naphtho[1,2-*c*:5,6-*c*′]bis[1,2,5]thiadiazol-5,10-diyl)-*alt*-(3,3′′′-di(2-octyldodecyl)-2,2′;5′,2′′;5′′,2′′′-quaterthiophen-5,5′′′-diyl)] (PNT4T-2OD); Fig. 1a) with significantly different polymer backbones but with similar arrangements of 2OD alkyl chains. Both PBTff4T-2OD and PNT4T-2OD exhibit significant temperature-dependent aggregation behaviour that leads to processing and morphology control and thus efficiency (including >10% for thick-film PSCs; Table 1, Supplementary Table 1 and Supplementary Fig. 7) comparable to those achieved by PffBT4T-2OD-based PSCs. R-SoXS and AFM studies confirmed that the polymer domain size of these two new polymers are similar to that of PffBT4T-2OD (30–40 nm). XRD characterization of PBTff4T-2OD:fullerene and PNT4T-2OD:fullerene films also showed strong (010) *π*–*π* stacking peaks that are similar to those observed for PffBT4T-2OD. Note that PNT4T-2OD also significantly outperforms its analogue polymer with 2-decyltetradecyl (2DT) alkyl chains^35^, providing another example that supports the critical importance of the size of the alkyl chains. The synthesis, characterization and device performance of PBTff4T-2OD and PNT4T-2OD are described in detail in the Supplementary Information (Supplementary Fig. 7, Table 1 and Supplementary Table 1).

Although second-position branched alkyl chains are a well-known structural motif and have been previously used on quaterthiophene-based polymers, previous work did not utilize a polymer with the most suitable alkyl chains nor were warm-casting methods used that optimally harnessed aggregation. They thus failed to reveal the connections between chemical structure, polymer aggregation during warm processing, morphology formation, polymer crystallinity and consequently PSC performance. Our study uncovered a new approach of aggregation and morphology control enabled by a structural feature (2OD alkyl chain) that is seemingly simple and commonly known, yet has surprisingly profound impact on PSC performances. The wide ranging applicability of our morphology control approach is supported by the three polymers and over 10 polymer:fullerene combinations that all yielded similar blend morphology and high-efficiency thick-film PSCs. Furthermore, the aggregation behaviour as observed by UV-Vis might serve as a useful screening tool to identify materials that yield good devices when cast from warm solutions.

Note that the chemical structures of the three donor polymers presented in the paper are distinctively different from previous state-of-the-art PTB7 family of polymers^12^,^13^,^17^. The PTB7 family polymers consist of an electron deficient fluorinated thieno[3,4-*b*]thiophene unit and a benzodithiophene unit with alkoxy, alkylthienyl or alkylthiothienyl substitution groups. The three polymers in this paper consist of an electron deficient unit (either difluorobenzothiadiazole or benzothiadiazole or naphthobisthiadiazole) combined with a quaterthiophene unit with two 2OD alkyl chains sitting on the first and fourth thiophenes. The difference in the chemical structures caused different aggregation properties, based on which different processing protocols are used. While PTB7 family polymers do not exhibit a strong temperature-dependent aggregation property and are often processed from room temperature solutions, the 4T-2OD based polymers are processed from warm solutions to utilize their temperature-dependent aggregation property so that the morphology and extent of molecular ordering can be explicitly controlled during casting.

To summarize, we report that exquisite control of aggregation results in high-performance thick-film PSCs for three different donor polymers and 10 polymer:fullerene combinations, all of which yielded efficiencies higher than the previous state of the art (9.5%). The common structural feature of the three donor polymers, the 2OD alkyl chains on quaterthiophene, causes a temperature-dependent aggregation behaviour that allows for the processing of the polymer solutions at moderately elevated temperature, and more importantly, controlled aggregation and strong crystallization of the polymer during the film cooling and drying process. This results in a near-ideal polymer:fullerene morphology (containing highly crystalline, preferentially orientated, yet small polymer domains) that is controlled by polymer aggregation during casting and thus insensitive to the choice of fullerenes. The branching position and size of the branched alkyl chains are critically important in enabling a well-controllable aggregation behaviour. Unnecessarily long alkyl chains (for example, 2DT) cause several detrimental effects including weaker laminar stacking, poorer absorption properties and less pure polymer domains. Our structural design rationales and aggregation and morphology control approach offer a new route to achieve high-performance thick-film PSCs that cannot be obtained from previous state-of-the-art material systems. Given that the field and record performance in the last few years has been mostly dominated by a single system (PTB7 family with PC_71_BM), the 10 material systems and three polymers based on a single and simple design feature presented here point to a plethora of possible materials combinations that should further improve the performance. Our approach will allow the PSC community to explore many more polymers and fullerene materials and to optimize their combinations (energy offsets, bandgap and so on) under a well-controlled morphological landscape that would greatly accelerate the materials and process development towards improved PSCs.

## Methods

### X-ray diffraction

XRD data were obtained from a PANanalytical XRD instrument (model name: Empyrean) using the parallel beam mode that is recommended by the instrument manufacturer to characterize thin-film samples. All XRD samples were spin cast on Si substrates to avoid strong scattering background of glass substrates. To rule out the effect of substrate properties on the crystallinity of polymer film samples, we also investigated polymer films on Si/ZnO substrates and found that the polymer films have similar scattering profiles (Supplementary Fig. 8) on these two types of substrates (Si/ZnO and Si). The polymer crystallinity is thus rather insensitive to the surface properties of the substrates. More details of XRD characterizations are provided in Supplementary Note 3.

### Cyclic voltammetry

Cyclic voltammetry was performed in an electrolyte solution of 0.1 M tetrabutylammonium hexafluorophosphate in acetonitrile, both working and counter electrodes were platinum electrode. Ag/AgCl electrode was used as the reference electrode; the Fc/Fc^+^ redox couple was used as an external standard (Supplementary Fig. 9 and Supplementary Table 6).

### UV-Vis absorption

UV-Vis absorption spectra were acquired on a Gary 50 UV-Vis spectrometer. All film samples were spin cast on ITO/ZnO substrates.

### Hole-only device

The hole mobility were measured using the SCLC method by using a device architecture of ITO/V_2_O_5_/PffBT4T-2OD (300 nm)/V_2_O_5_/Al by taking current–voltage curves and fitting the results to a space charge limited form, where the SCLC is described by:


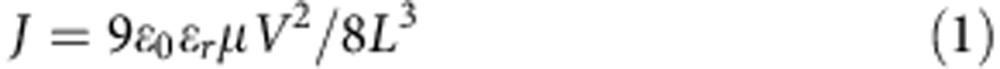


Where *ε*_0_ is the permittivity of free space, *ε*_r_ is the dielectric constant of the polymer, *μ* is the hole mobility, *V* is the voltage drop across the device and *L* is the thickness of the polymer. The dielectric constant *ε*_r_ is assumed to be ~3, which is a typical value for conjugated polymers.

### GIWAXS characterization

GIWAXS measurements were performed at beamline 7.3.3 at the Advanced Light Source (ALS)^39^. Samples were prepared on Si substrates using identical blend solutions as those used in devices. The 10 keV X-ray beam was incident at a grazing angle of 0.11°–0.15°, which maximized the scattering intensity from the samples. The scattered X-rays were detected using a Dectris Pilatus 1 M photon counting detector.

### Resonant soft X-ray scattering

R-SoXS transmission measurements were performed at beamline 11.0.1.2 at the ALS^30^. Samples for R-SoXS measurements were prepared on a PSS modified Si substrate under the same conditions as those used for device fabrication, and then transferred by floating in water to a 1.5 × 1.5 mm, 100-nm thick Si_3_N_4_ membrane supported by a 5 × 5 mm, 200 μm thick Si frame (Norcada Inc.). Two dimensional scattering patterns were collected on an in-vacuum CCD camera (Princeton Instrument PI-MTE). The beam size at the sample is ~100 μm by 200 μm. The composition variation (or relative domain purity) over the length scales probed can be extracted by integrating scattering profiles to yield the total scattering intensity. The purer the average domains are, the higher the total scattering intensity. Owing to a lack of absolute flux normalization, the absolute composition cannot be obtained by only R-SoXS. In order to get a sense of how pure the domains are, we annealed the PffBT4T-2OD/fullerene blend at 130 °C for different length of time, 0, 10, 20, 40 and 120 min. The unannealed sample exhibits very pure domains, that is, almost 90% of the saturated value.

### AFM characterization

AFM measurements were performed by using a Scanning Probe Microscope-Dimension 3100 in tapping mode. All film samples were spin casted on ITO/ZnO substrates.

### Photoluminescence quenching measurements

Photoluminescence spectra were measured on samples on ITO/ZnO substrates upon excitation of a 671-nm laser beam. The PL quenching efficiency of PffBT4T-2OD was estimated from the ratio of the PL intensity of a PffBT4T-2OD:fullerene film sample to that of the PffBT4T-2OD control sample. (Supplementary Fig. 10)

### Solar cell fabrication and testing

Pre-patterned ITO-coated glass with a sheet resistance of ~15 Ω per square was used as the substrate. It was cleaned by sequential sonications in soap DI water, DI water, acetone and isopropanol for 15 min at each step. After ultraviolet/ozone treatment for 60 min, a ZnO electron transport layer was prepared by spin coating at 5,000 r.p.m. from a ZnO precursor solution (diethyl zinc). Active layer solutions (D/A ratio 1:1.2) were prepared in CB/DCB (1:1 volume ratio) with or without 3% of DIO (polymer concentration: 9 mg ml^−1^). To completely dissolve the polymer, the active layer solution should be stirred on a hot plate at 110 °C for at least 3 h. Before spin coating, both the polymer solution and ITO substrate are preheated on a hot plate at ~110 °C. Active layers were spin coated from the warm polymer solution on the preheated substrate in a N_2_ glovebox at 800 r.p.m. to obtain thicknesses of ~300 nm. (The spin casting of high-performance PSC films is described in Supplementary Note 4 in details. The processing of PNT4T-2OD also requires the use of a metal chuck as described in Supplementary Note 4. High-temperature and high spin rate samples are described in Supplementary Note 5). The polymer/fullerene films were then annealed at 80 °C for 5 min before being transferred to the vacuum chamber of a thermal evaporator inside the same glovebox. At a vacuum level of 3 × 10^−6^ Torr, a thin layer (20 nm) of MoO_3_ or V_2_O_5_ was deposited as the anode interlayer, followed by deposition of 100 nm of Al as the top electrode. All cells were encapsulated using epoxy inside the glovebox. Device *J*–*V* characteristics was measured under AM1.5G (100 mW cm^−2^) using a Newport solar simulator. The light intensity was calibrated using a standard Si diode (with KG5 filter, purchased from PV Measurement) to bring spectral mismatch to unity. *J*–*V* characteristics were recorded using a Keithley 236 source meter unit. Typical cells have devices area of ~5.9 mm^2^, which is defined by a metal mask with an aperture aligned with the device area. EQEs were characterized using a Newport EQE system equipped with a standard Si diode. Monochromatic light was generated from a Newport 300 W lamp source. One of our best cells was sent to an accredited solar cell calibration laboratory (Newport Corporation) for certification, confirming an efficiency of 10.36±0.22%, with *V*_OC_=0.7743±0.0077 V, *I*_SC_=0.00079±0.00001 A, area=0.0425±0.0001, cm^2^, FF=72.0±1.5 (Supplementary Fig. 11).

## Author contributions

Y.L. synthesized PffBT4T-2OD; J.Z. designed PNT4T-2OD, synthesized 5,10-Dibromonaphtho[1,2-c:5,6-c']bis[1,2,5]thiadiazole and carried out AFM measurements; Z.L. synthesized PBTff4T-2OD; J.Z., H.L., Y.L., H.H. and Z.L. synthesized fullerenes; C.M., H.H., K.J. and H.Y. fabricated and tested PSC devices; W.M. carried out GIWAXS and R-SoXS measurements and analysis; K.J. carried out XRD analysis; H.L. synthesized PNT4T-2OD; H.A. supervised GIWAXS and R-SoXS work, and helped design experimental protocols; H.Y. conceived and directed the project; H.A. and H.Y. wrote the paper with input from all authors who reviewed the final paper.

## Additional information

**How to cite this article:** Liu, Y. *et al.* Aggregation and morphology control enables multiple cases of high-efficiency polymer solar cells. *Nat. Commun.* 5:5293 doi: 10.1038/ncomms6293 (2014).

## Supplementary Material

Supplementary Information Supplementary Figures 1-11, Supplementary Tables 1-6, Supplementary Notes 1-5, Supplementary Methods and Supplementary References

## Figures and Tables

**Table 1:** PSC performance of 10 high-efficiency polymer:fullerene material combinations.

**Active layer**	***V***_**OC**_ **(V)**	***J***_**SC**_ **(mA cm**^**−2**^**)**	**FF**	**PCE (%)**
PffBT4T-2OD:TC_71_BM	0.77	18.8	0.75	10.8 (10.3)*
PffBT4T-2OD:PC_71_BM	0.77	18.4	0.74	10.5 (10.2)
PffBT4T-2OD:PC_61_PM	0.77	17.7	0.76	10.4 (10.1)
PffBT4T-2OD:ICMA	0.78	16.4	0.77	9.8 (9.4)
PffBT4T-2OD:TC_61_PM	0.75	17.4	0.74	9.7 (9.3)
PffBT4T-2OD:PC_61_BM	0.77	17.1	0.73	9.6 (9.3)
PBTff4T-2OD:PC_71_BM	0.77	18.2	0.74	10.4 (10.0)
PBTff4T-2OD:TC_71_BM	0.76	18.7	0.68	9.7 (9.4)
PBTff4T-2OD:PC_61_PM	0.76	18.6	0.69	9.6 (9.2)
PNT4T-2OD:PC_71_BM	0.76	19.8	0.68	10.1 (9.7)

FF, fill factor; PCE, power conversion efficiency; PSC, polymer solar cell.

^*^The values in parentheses stand for the average PCEs from over 20 devices for PffBT4T-2OD and from over 10 devices for PBTff4T-2OD and PNT4T-2OD.
