# Supplementary material for: Aggregation and morphology control enables multiple cases of high-efficiency polymer solar cells
Source: Nat Commun. 2014 Nov 10;5:5293. doi: 10.1038/ncomms6293 (PMC4242436; doi:10.1038/ncomms6293)
Supplement: Supplementary Information — Supplementary Figures 1-11, Supplementary Tables 1-6, Supplementary Notes 1-5, Supplementary Methods and Supplementary References [file ncomms6293-s1.pdf]

Supplementary Figures

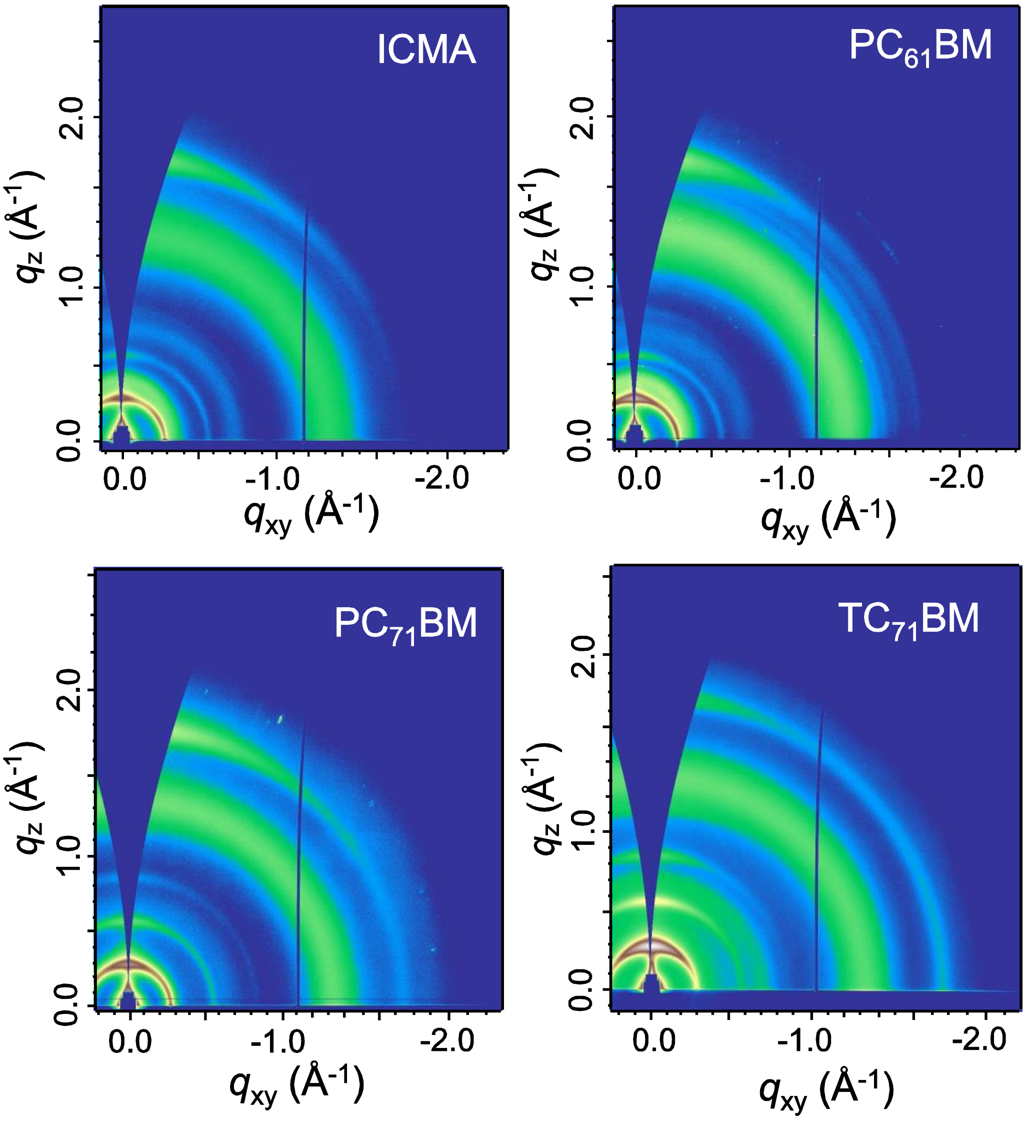

Supplementary Figure 1. GIWAXS 2D patterns of PffBT4T-2OD:fullerene blends. **a,** PffBT4T-2OD:ICMA; **b,** PffBT4T-2OD:$PC_{61}BM$; **c,** PffBT4T-2OD:$PC_{71}BM$; **d,** PffBT4T-2OD:$TC_{71}BM$.

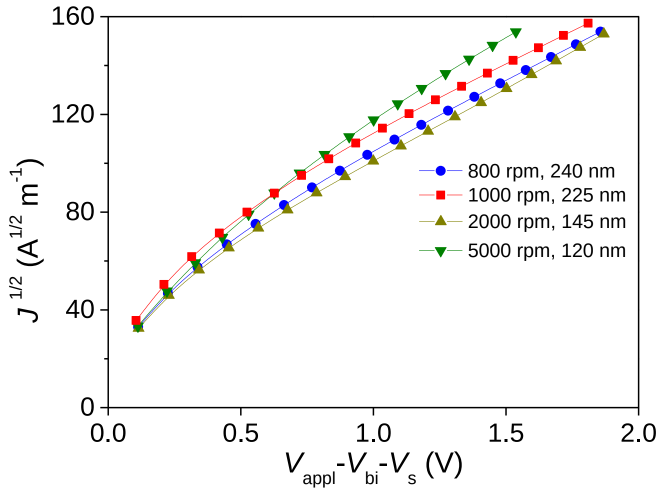

Supplementary Figure 2. *J-V* curves of the hole-only device of PffBT4T-2OD:PC_{61}PM fabricated at various spin rates.

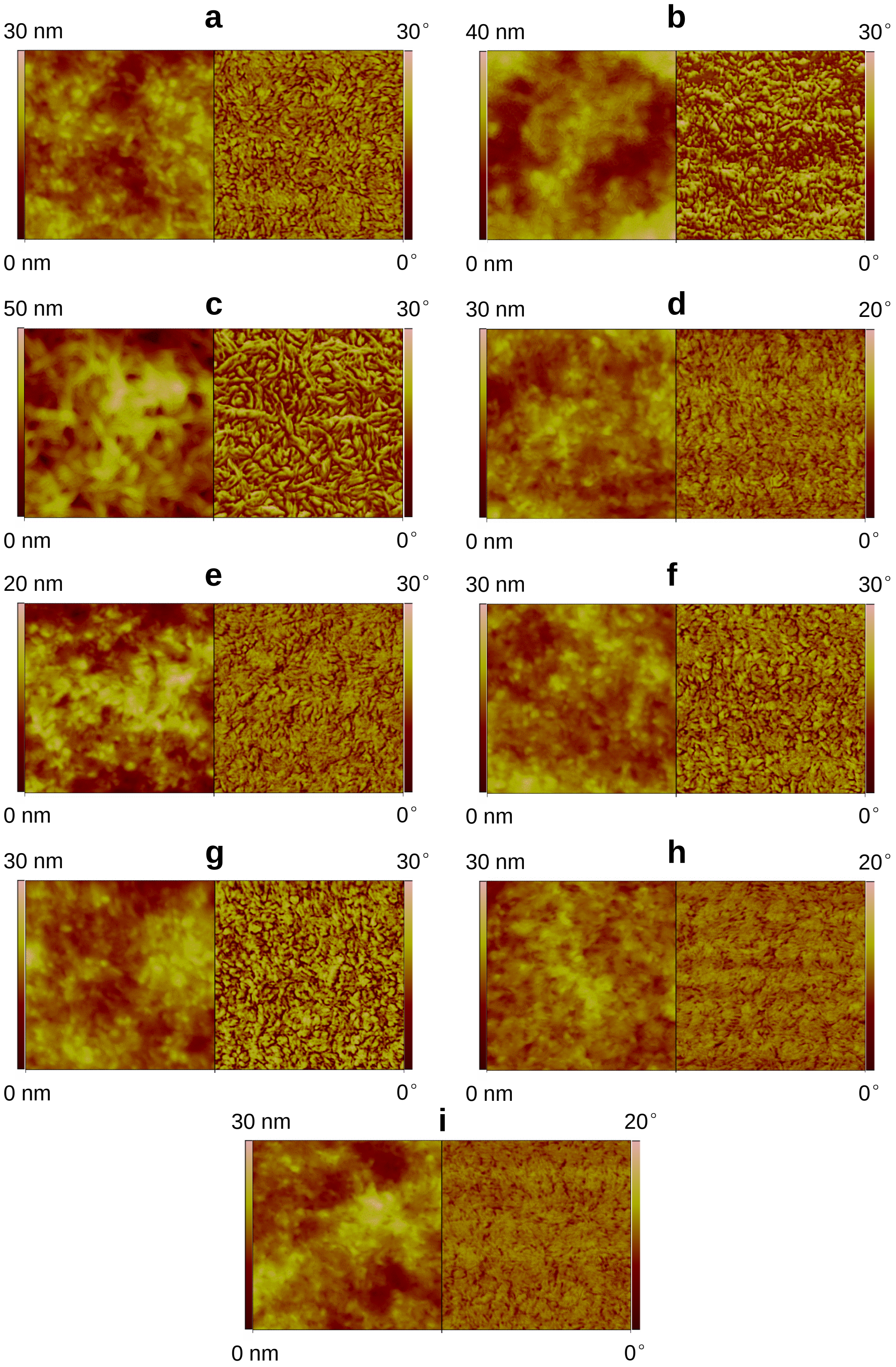

Supplementary Figure 3. AFM (1×1 μm) images of PffBT4T-2OD:fullerene blend films. The fullerenes are **a,** $TC_{71}BM$; **b,** $PC_{71}BM$; **c,** $PC_{61}PM$; **d,** ICMA; **e,** $TC_{61}PM$; **f,** NCMM; **g,** ICMM; **h,** MOPFP; **i,** NCMA. The height and phase images are displayed on the left and right sides, respectively.

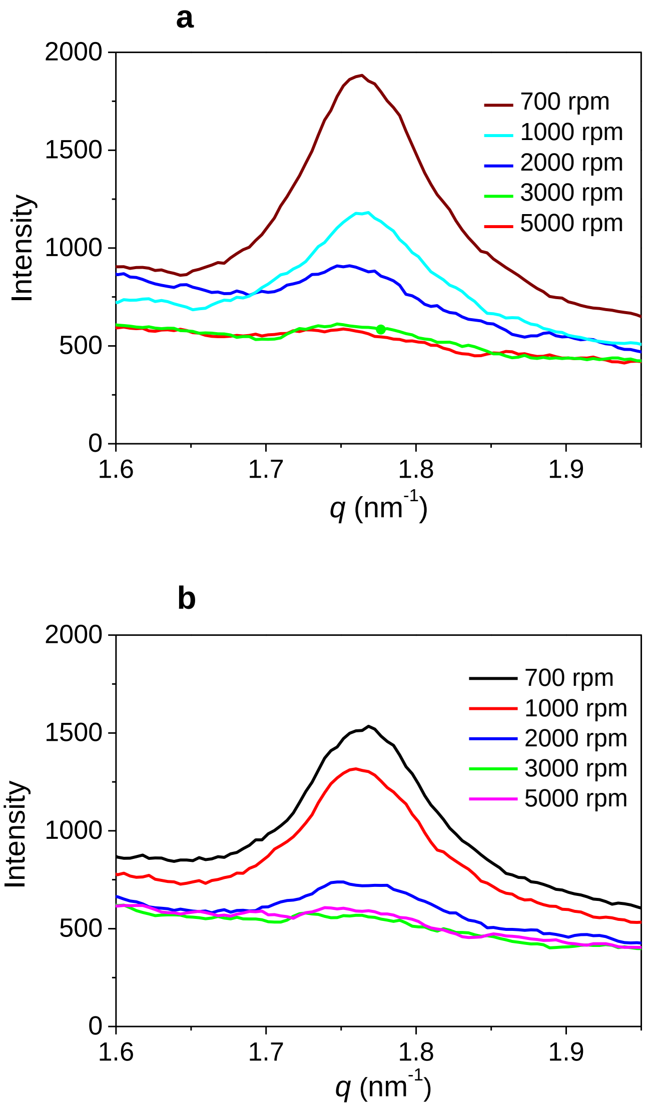

Supplementary Figure 4. Intensity of (010) diffraction peaks (obtained from XRD). a, PffBT4T-2OD:PC$_{61}$PM blend films; **b,** PffBT4T-2OD:PC$_{61}$BM blend films spun at different spinrates and a solution/substrate temperature of ~70 °C.

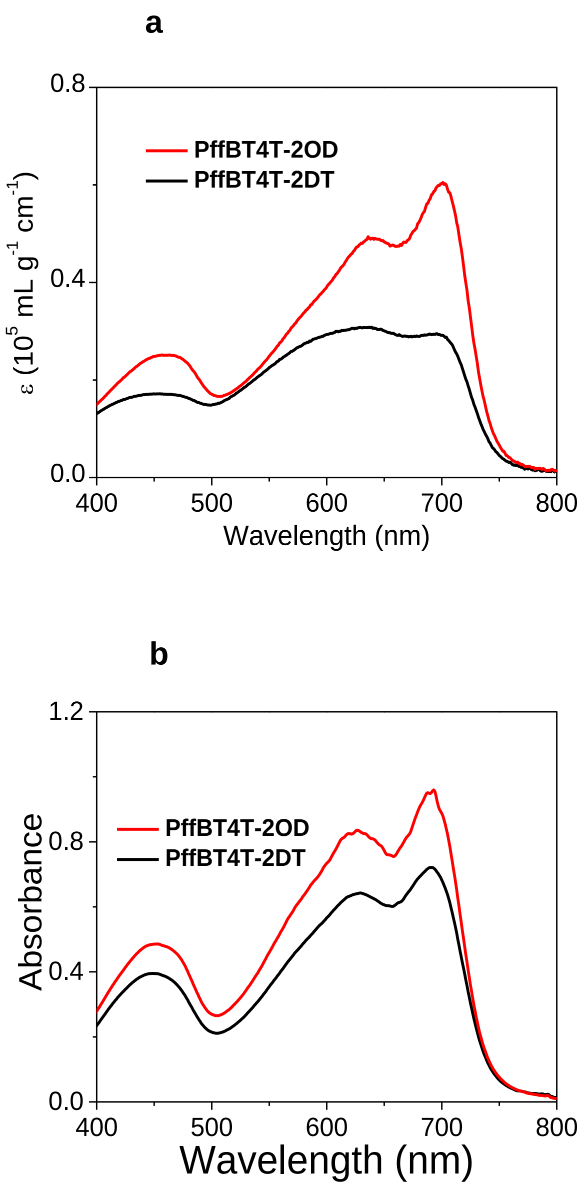

Supplementary Figure 5. Optical characterization of PffBT4T-2OD and PffBT4T-2DT. a, UV-Vis absorption coefficients in solution; **b,** comparison of the optical absorbance of pure films normalized by thickness.

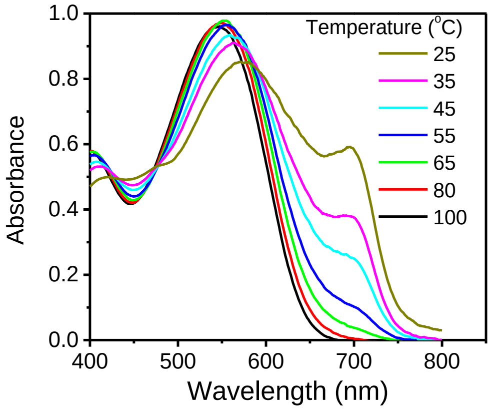

Supplementary Figure 6. UV-Vis spectra of low molecular weight (M_n = 16.6 kDa, M_w = 29.5 kDa) PffBT4T-2OD solution at temperatures as indicated.

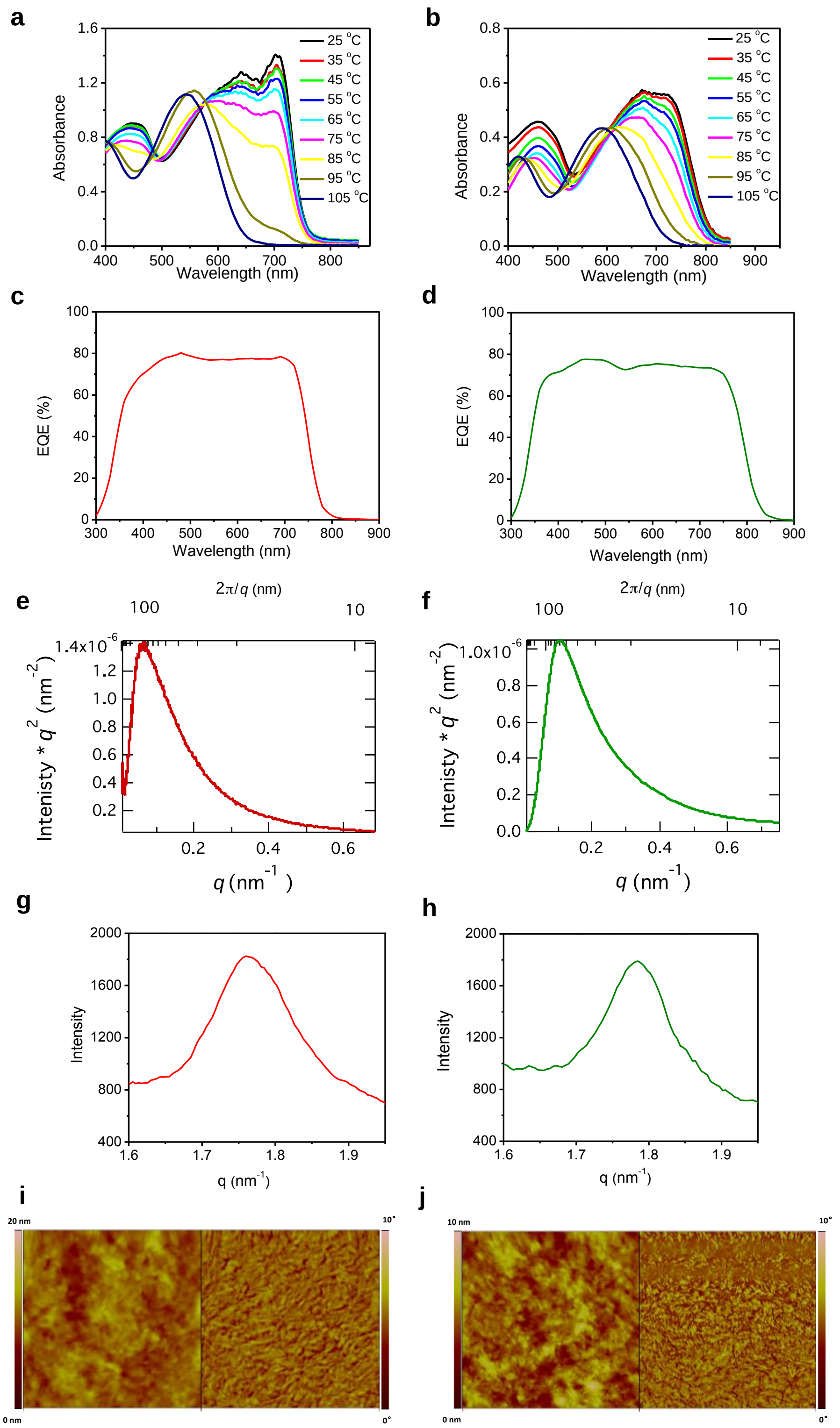

Supplementary Figure 7. Characterization of PBTff4T-2OD and PNT4T-2OD. UV spectra of **a.** PBTff4T-2OD and **b.** PNT4T-2OD at various temperatures in 0.02 mg/mL *o*-DCB solutions. EQE spectra of **c.** PBTff4T-2OD:$PC_{71}BM$ PSCs; **d.** PNT4T-2OD:$PC_{71}BM$ PSCs. R-SoXS profile of **e.** a PBTff4T-2OD:$PC_{71}BM$ film; **f.** a PBTff4T-2OD:$PC_{71}BM$ film. (010) diffraction peaks (obtained by XRD) of **g.** PBTff4T-2OD:$PC_{71}BM$ films; **h.** PNT4T-2OD:$PC_{61}BM$ films. AFM (1×1 μm) images of **i.** a PBTff4T-2OD:$PC_{71}BM$ blend film; **j.** a PNT4T-2OD:$PC_{71}BM$ blend film.

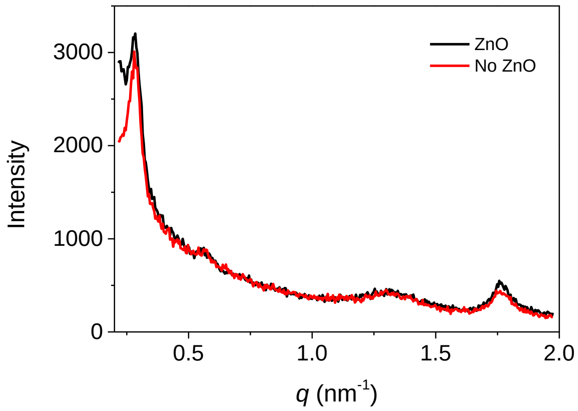

Supplementary Figure 8. Scattering profiles (obtained from XRD) of PffBT4T-2OD:$PC_{71}BM$ blend films spun on Si substrates with or without ZnO.

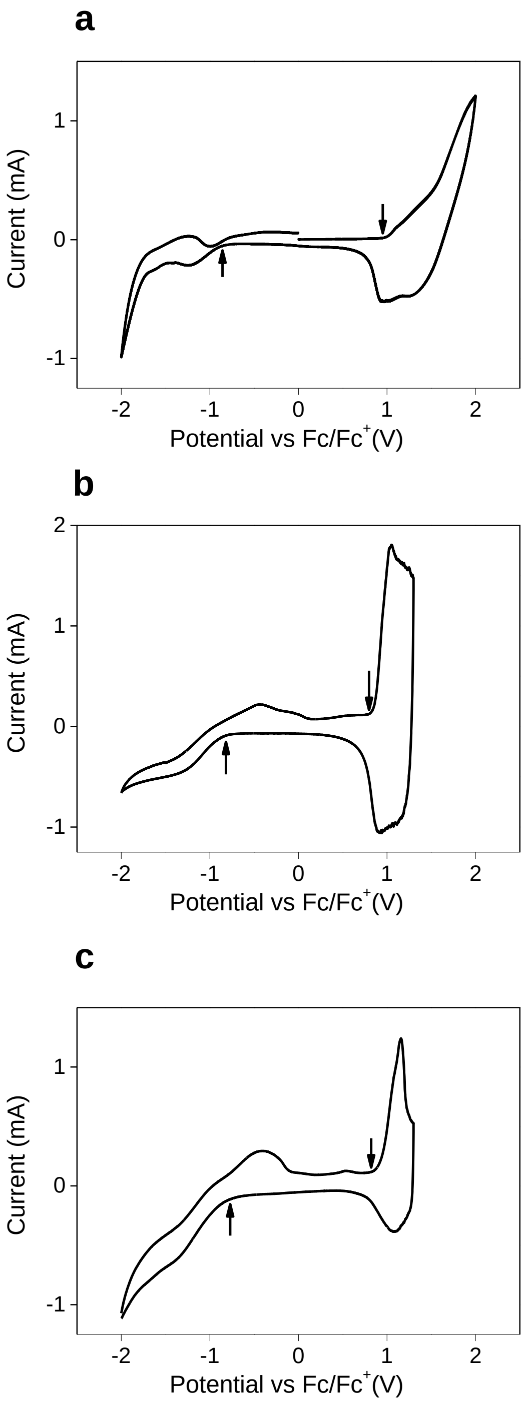

Supplementary Figure 9. Electrochemical cyclic voltammograms. a, PffBT4T-2OD; **b**, PBTff4T-2OD **c**, PNT4T-2OD. The arrows indicate the potential onset of the oxidation or reduction reactions in the electrochemical measurements.

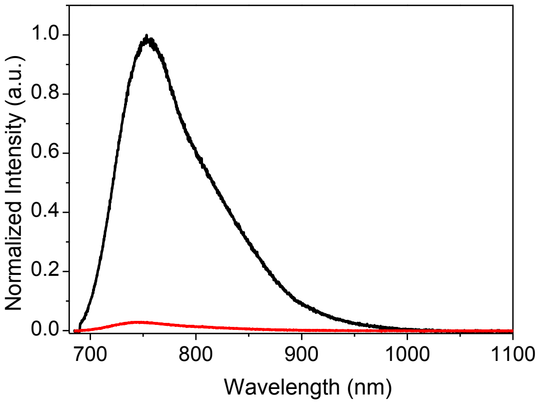

Supplementary Figure 10. The PL spectrum of a PffBT4T-2OD:$PC_{71}BM$ (red line) film sample compared with the PL spectrum of a PffBT4T-2OD control sample (black line). The peak intensity of the blend sample is about 3% of that of the control sample.

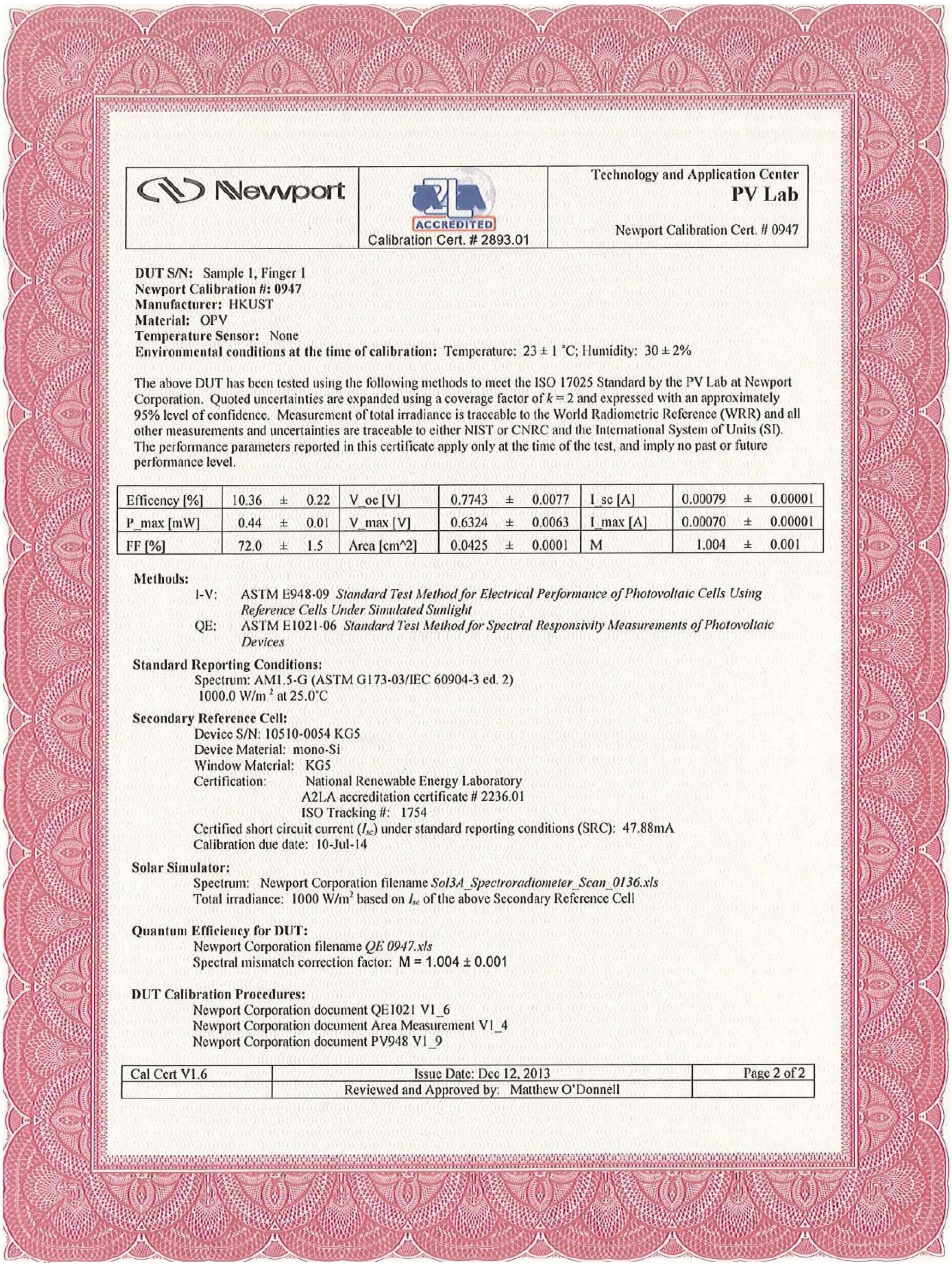

Newport	A2LA ACCREDITED Calibration Cert. # 2893.01	Technology and Application Center<br>PV Lab<br>Newport Calibration Cert. # 0947

DUT S/N: Sample 1, Finger 1
Newport Calibration #: 0947
Manufacturer: HKUST
Material: OPV
Temperature Sensor: None
Environmental conditions at the time of calibration: Temperature: 23 ± 1 °C; Humidity: 30 ± 2%

The above DUT has been tested using the following methods to meet the ISO 17025 Standard by the PV Lab at Newport Corporation. Quoted uncertainties are expanded using a coverage factor of $k = 2$ and expressed with an approximately 95% level of confidence. Measurement of total irradiance is traceable to the World Radiometric Reference (WRR) and all other measurements and uncertainties are traceable to either NIST or CNRC and the International System of Units (SI). The performance parameters reported in this certificate apply only at the time of the test, and imply no past or future performance level.

Efficency [%]	10.36 ± 0.22	V_oc [V]	0.7743 ± 0.0077	I_sc [A]	0.00079 ± 0.00001
P_max [mW]	0.44 ± 0.01	V_max [V]	0.6324 ± 0.0063	I_max [A]	0.00070 ± 0.00001
FF [%]	72.0 ± 1.5	Area [cm^2]	0.0425 ± 0.0001	M	1.004 ± 0.001

Methods:
I-V: ASTM E948-09 *Standard Test Method for Electrical Performance of Photovoltaic Cells Using Reference Cells Under Simulated Sunlight*
QE: ASTM E1021-06 *Standard Test Method for Spectral Responsivity Measurements of Photovoltaic Devices*

Standard Reporting Conditions:
Spectrum: AM1.5-G (ASTM G173-03/IEC 60904-3 ed. 2)
1000.0 W/m ^{2} at 25.0°C

Secondary Reference Cell:
Device S/N: 10510-0054 KG5
Device Material: mono-Si
Window Material: KG5
Certification: National Renewable Energy Laboratory
A2LA accreditation certificate # 2236.01
ISO Tracking #: 1754
Certified short circuit current (I_{sc}) under standard reporting conditions (SRC): 47.88mA
Calibration due date: 10-Jul-14

Solar Simulator:
Spectrum: Newport Corporation filename *Sol3A_Spectroradiometer_Scan_0136.xls*
Total irradiance: 1000 W/m^2 based on I_{sc} of the above Secondary Reference Cell

Quantum Efficiency for DUT:
Newport Corporation filename *QE 0947.xls*
Spectral mismatch correction factor: M = 1.004 ± 0.001

DUT Calibration Procedures:
Newport Corporation document QE1021 V1_6
Newport Corporation document Area Measurement V1_4
Newport Corporation document PV948 V1_9

Cal Cert V1.6	Issue Date: Dec 12, 2013	Page 2 of 2
	Reviewed and Approved by: Matthew O'Donnell	

Supplementary Figure 11. Independent certification in Newport Corporation confirming a power conversion efficiency of 10.36%.

Supplementary Tables

Supplementary Table 1. Performance of PSCs based on PffBT4T-2OD and PNT4T-2OD with various fullerenes.

Active layer	V_{OC} [V]	J_{SC} [mA cm^{-2}]	FF	PCE [%]
PNT4T-2OD:PC_{61}PM	0.73	18.8	0.67	9.3 (8.9)
PffBT4T-2OD:NCMM	0.76	17.7	0.70	9.4 (9.3)
PffBT4T-2OD:ICMM	0.78	17.0	0.70	9.3 (9.1)
PffBT4T-2OD:MOPFP	0.76	17.2	0.70	9.2 (9.1)
PffBT4T-2OD:PC_{61}BE	0.78	17.1	0.69	9.2 (8.9)
PffBT4T-2OD:NCMA	0.77	17.7	0.67	9.1 (8.3)
PffBT4T-2OD:ICEM	0.80	16.5	0.67	8.9 (8.7)
PffBT4T-2OD:PC_{61}MM	0.75	16.8	0.70	8.9 (8.5)
PffBT4T-2OD:TFP	0.76	16.9	0.68	8.8 (8.6)
PffBT4T-2OD:PFP	0.77	16.4	0.68	8.6 (8.3)
PffBT4T-2OD:TC_{61}BM	0.77	17.5	0.64	8.6 (8.3)

Supplementary Table 2. (010) Coherence length of and integration of (010) peak intensity (normalized by thickness) for PffBT4T-2OD and PffBT4T-2OD:fullerene films spun at different rates (data obtained from laboratory-based XRD).

Spinrate [rpm]		700	1000	2000	3000	5000
**PffBT4T-2OD (Pure)**	(010) Coherence Length [nm]	5.7	5.2	3.5	1.0	0.9
	Integration of (010) peak	32	24	21	16	9
**PffBT4T-2OD:PC_{61}PM**	(010) Coherence Length [nm]	6.6	6.5	2.9	2.8	2.4
	Integration of (010) peak	61	45	24	11	9
**PffBT4T-2OD:PC_{61}BM**	(010) Coherence Length [nm]	6.6	6.3	4.4	1.3	1.2
	Integration of (010) peak	37	38	27	12	9

Supplementary Table 3. Hole mobility of PffBT4T-2OD:PC_{61}PM blend films spun at different spin rates (hole mobility calculated based on the space-charge-limited-current of hole-only devices).

Spinrate [rpm]	Thickness [nm]	Mobility [cm^2 V^{-1} s^{-1}]
800	240	1.7×10^{-2}
1000	225	1.3×10^{-2}
2000	145	3.7×10^{-3}
5000	120	3.1×10^{-3}

Supplementary Table 4. Performance of PffBT4T-2OD:PC_{61}PM-based PSC devices fabricated at high spinrates and high temperatures. (In order to obtain similar film thicknesses for a fair comparison, high spinrate/high temperature devices were spun from a more concentrated solution (14 mg/mL), while the control device (800 rpm/70 °C) was spun from a 9 mg/mL solution in 1:1 CB/*o*-DCB containing 3% of DIO.)

**Spinrate [rpm]**	**Substrate Temperature [°C]**	**V_{OC} [V]**	**J_{SC} [mA cm^{-2}]**	**FF**	**PCE [%]**	**Thickness [nm]**
5000	110	0.81	7.6	0.59	3.6	260
3000	90	0.80	10.0	0.65	5.2	251
800	70	0.78	17.5	0.75	10.2	300

Supplementary Table 5. PSC performances of PffBT4T-2OD batches with different molecular weights combined with PC_{71}BM.

**M_n [kDa]**	**M_w [kDa]**	**V_{OC} [V]**	**J_{SC} [mA cm^{-2}]**	**FF**	**PCE [%]**
16.6	29.5	0.72	16.7	0.63	7.7
38.9	71.7	0.76	17.9	0.65	9.0
47.5	93.7	0.77	18.6	0.73	10.4

Supplementary Table 6. HOMO Level (measured by cyclic voltammetry), optical bandgap (estimated based on film absorption onset), and LUMO level (calculated based on HOMO and optical bandgap) of PffBT4T-2OD, PBTff4T-2OD, and PNT4T-2OD.

	**PffBT4T-2OD**	**PBTff4T-2OD**	**PNT4T-2OD**
HOMO $_{CV}$ [eV]	-5.34	-5.20	-5.24
Bandgap [eV]	1.65	1.63	1.53
LUMO $_{Opt.}$ [eV]	-3.69	-3.57	-3.71

Supplementary Notes

Supplementary Note 1. Comparison between PffBT4T-2OD and PffBT4T-1ON, PffBT4T-3OT. For the analogue polymers, it is highly challenging to synthesize PffBT4T-3OT with higher molecular weight (MW) than what we obtained (M_w = 36 kDa). The reason is because that PffBT4T-3OT has much lower solubility and stronger aggregation than PffBT4T-2OD. During the synthesis of PffBT4T-3OT, once the MW reached a certain level, PffBT4T-3OT polymer solution formed a complete gel that became a precipitated solid polymer even at 130 °C in the reaction vessel. This makes it challenging to further increase the MW of the polymers. The lower MW of PffBT4T-3OT compared to PffBT4T-2OD should not change the conclusion of our comparisons. As the shown batch of PffBT4T-3OT already exhibited much stronger aggregation than PffBT4T-2OD, higher MW versions of PffBT4T-3OT would have even stronger aggregation. In the case of PffBT4T-1ON, the M_w is 52.2 kDa, which is lower than that of the best-performing batches of PffBT4T-2OD. To rule out the possibility that the lower MW of PffBT4T-1ON is the main reason for its weak aggregation property, we also compared the UV-Vis spectra of PffBT4T-1ON with lower MW (M_w = 29.5 kDa) PffBT4T-2OD (Supplementary Figure 6). The comparison supports that the branching position of PffBT4T-1ON's alkyl chains is a more important reason that causes the dramatic difference in the aggregation properties of the PffBT4T-2OD and PffBT4T-1ON. Similar comparison was also done for PffBT4T-3OT and PffBT4T-2OD with similar molecular weights. The dramatic difference in the UV-Vis absorption and aggregation behaviors of PffBT4T-1ON and PffBT4T-3OT from PffBT4T-2OD caused extremely poor PSC device performance. This shows that the branching position of the alkyl chain is the most important structural feature among the various polymer structures we studied and it has dramatic impacts on PSC performance.

The dramatic blue-shift of absorption spectra of PffBT4T-1ON (compared with PffBT4T-2OD) indicates that there is significant twisting between two the adjacent thiophene rings, between which the 1st position branched alkyl chains of PffBT4T-1ON are sitting. It is well known that the two adjacent head-to-head straight alkyl chains in regio-random P3HT can introduce significant steric hindrance effect and cause severe twisting between the two adjacent thiophenes. In the case of PffBT4T-1ON, although there is only one alkyl chain sitting between two adjacent thiophenes, the very bulky 1st position branched alkyl chains apparently causes a similar steric hindrance effect and thus a dramatic blue-shift in the absorption spectra of the polymer. For PffBT4T-2OD and PffBT4T-3OT, as the branching position is moved away from the polymer backbone, the steric hindrance effect caused by the very bulky branched alkyl group decreases significantly. Our comparative study indicates that PffBT4T-1ON's alkyl chains cause too much steric hindrance, which results in poor aggregation at both 25 °C and 85 °C in solution and thus low crystallinity in the film. PffBT4T-3OT's alkyl chains provide too little steric hindrance, which makes aggregation of PffBT4T-3OT too strong even at 85 °C and

makes it extremely difficult to process. PffBT4T-2OD's 2nd position branched alkyl chains offer an optimal tradeoff that allows for controllable aggregation of PffBT4T-2OD during the film-forming process to form a near-ideal morphology containing highly crystalline, sufficiently pure, yet small polymer domains.

Supplementary Note 2. Impacts of polymer molecular weight on PSC performance. Our study also demonstrates that the MW of polymers is important to obtain optimal aggregation and the best PSC performance. In the case of PffBT4T-2OD, higher MW (M_n = 47.5 kDa, M_w = 93.7 kDa) polymers exhibit stronger tendency of aggregation and higher efficiency than lower MW (M_n = 16.6 kDa, M_w = 29.5 kDa) ones (Figure 1d, Supplementary Figure 6, Supplementary Table 5). The weaker aggregation of low MW PffBT4T-2OD is consistent with the lower intensity of 0-0 transition peak in the UV-Vis absorption spectrum of PffBT4T-2OD'solution at 25 °C (Supplementary Figure 6). In addition, lower MW versions of PffBT4T-2OD can be partially or completely dissolved in chloroform and their NMR sepctra can be taken in a chloroform solution at room temperature. In contrast, the higher MW PffBT4T-2OD is not soluble even in hot chloroform and only soluble in hot chlorobenzene or tetrachloroethane. The solution of high MW PffBT4T-2OD completely gels at room temperature, therefore, its NMR spectrum needs to be acquired in a d_2-tetrachloroethane solution at above 100 °C.

In the literature, there were structurally similar polymers with lower MW and thus lower PSC performance. Ng and coworkers reported a low MW (M_n = 9.8 kDa, M_w = 17 kDa) polymer (PFBT-T20TT) that achieved a PSC efficiency of 6.3%. [2] Note that the low MW polymer Ng reported can be well dissolved in chloroform as their NMR data were taken in a chloroform solution at room temperature. Especially considering that the polymer by Ng and coworkers has a number average molecular weight of only 9.8 kDa, meaning the polymer batch contains a significant amount of oligomers with less than 10 repeating units. Such low MW oligomers have significant negative impacts on PSC performances. There is another reported PffBT4T-2OD analogue polymer in the literature (PTh_4FBT),[3] which can achieve 6.82% efficiency for a thin-film (95 nm) PSC. Unfortunately, this report did not provide any MW data, which is critically important in understanding the aggregation behavior of the polymer and comparing that polymer with other structurally similar polymers. The fact that polymer is partially soluble in chloroform and that the NMR spectrum of the polymer was taken from a chloroform solution at room temperature indicates that the polymer solution does not gel at room temperature. Therefore, it is reasonable to assume the polymer reported does not have sufficiently high MW to enable PffBT4T-2OD's aggregation behavior that is described in this paper. This assumption of low MW of the polymer in that report[3] has also been pointed out by Chen and coworkers.[4] We have attempted to synthesize these two polymers with higher MW and found there are various synthetic challenges (e.g., the monomer of PTh_4FBT is difficult to purify) to obtain polymers with high MW. Although we are not able to obtain PFBT-T20TT and PTh_4FBT with MWs as high as the best PffBT4T-2OD batch, our preliminary attempts have yielded polymers with intermediate MWs (M_w of 30-40 kDa) and thus

better efficiencies (8-9%) than these previous reports. The study on these additional polymers requires extensive synthetic work to obtain ideal polymer MW and is beyond the scope of this paper.

Supplementary Note 3. Characterizations of (010) peaks of PffBT4T-2OD:fullerene films by XRD. Our GIWAXS data of PffBT4T-2OD show that the (010) intensity is more significant in the out-of plane direction than the in-plane direction, indicating a strong preferential face-on orientation relative to the electrodes that is favourable for charge transport in the direction across the active layer. Due to this preferential face-on orientation, the (010) diffraction peak can also be clearly observed with standard XRD characterization. Therefore, we have carried out extensive XRD experiments to characterise the crystallinity of PffBT4T-2OD:fullerene films processed at various conditions. This comprehensive study allowed us to understand the impact of PffBT4T-2OD's aggregation behavior on the crystallinity of PffBT4T-2OD. Note that the highest (010) coherence length values of PffBT4T-2OD:fullerene films calculated based on an laboratory XRD are about 6.5 nm, which is slightly lower than the coherence length values (8.5 nm) we obtained based on GIWAXS data. While GIWAXS uses a highly monochromic and highly focused X-ray beam, the X-ray beam of an XRD instrument is not sufficiently monochromic or highly focused. Consequently, the (010) diffraction peak by XRD is broadened compared to that from GIWAXS, leading to smaller apparent coherence lengths. To confirm this, we have measured the same sample by XRD and GIWAXS and found the coherence length values obtained were 5.2 nm and 8.5 nm, respectively. The coherence length data presented in the paper are based on raw XRD data and are not corrected for the factors described above. The XRD equipment limitation does not invalidate the systematic trends we observed and thus XRD yields valid qualitative comparative evaluations. Furthermore, the intensity comparisons made are entirely unaffected by instrumental resolution limitations.

Generally, we spincast PffBT4T-2OD:fullerene solutions on a Si substrate for XRD experiment. A small incident angle (0.5 degree) was used and the diffraction intensity was recorded with a detector scan. At this angle, the X-rays penetrate the film completely. The (010) diffraction peak is observed at an angle of ~24-25 degree. The diffraction profiles are fitted by a Gaussian function to obtain the full width at half maximum (FWHM) of the width. The coherence length was calculated based on FWHM following literature procedure.[1] While the (010) coherence length can tell information about the length of the π-π stacked distance for polymer chains, the integration of (010) peaks can provide information on the relative amount of crystalline polymer domains in the film. As different processing conditions may yield films with different thicknesses, the integrated values (Supplementary Table 2) of the (010) diffraction peaks are corrected for the thickness differences between the samples in the same series.

Supplementary Note 4. Processing temperatures and procedures of PffBT4T-2OD.

At an elevated temperature of 85 °C, PffBT4T-2OD is disaggregated and can be well dissolved in chlorobenzene at a concentration of 8-10 mg/mL. When this solution cools down to room temperature, it becomes a complete gel. Therefore, PffBT4T-2OD or PffBT4T-2OD:fullerene films are always cast from warm solutions (60-80 °C) onto a substrate initially at the same temperature. During the spincasting process, as the solvent evaporates and the temperature of the substrate decreases, PffBT4T-2OD can strongly aggregate and form highly crystalline and pure polymer domains. To cast a warm solution of PffBT4T-2OD:fullerene blend, both the solution and ITO substrate are pre-heated to about 100-110 °C before casting. Then, the substrate is transferred onto the spincoater and the warm solution is added onto the substrate. There is typically a time delay from the point when the substrate is removed from the hotplate to the start of spincasting. During this time, the substrate cools to some extent. Consequently, the temperature of the substrate was measured right before the spincasting and the measured temperature was used as the reference temperature in our discussion and in Figure 2e. The optimal temperature to preheat the substrate and solution is about 100-110 °C, which leads to a substrate temperature of about 60-70 °C right before the starting point of the spincasting. This gives a reasonably large and easy to access temperature window to process PffBT4T-2OD, aiding in achieving high-efficiency PSCs reproducibly. The optimized spinrates and solution concentrations used are typically about 800 rpm and 8-10 mg/mL with 3% of DIO additive. These conditions are used to prepare the samples described in this paper unless otherwise stated. In some cases, higher substrate temperatures are employed to understand the impact of substrate temperatures on PffBT4T-2OD's crystallinity. For example, for the case of 110 °C–processed film in Figure 2e, the substrate needs to be preheated on a 130 °C hotplate. In addition, to slow down the cooling of the substrate during the operation, the substrate was attached to a metal chuck that was preheated together with the substrate and that served as a "heat reservoir" to slow down the cooling of the substrate before the start of spincasting.

Supplementary Note 5. Performance of PffBT4T-2OD-based PSCs processed at high spinrates and high substrate temperatures. We also fabricated PSC devices using high temperature and high spinrate conditions to understand the correlation between process conditions, polymer crystallinity, UV-Vis absorption spectrum and the performance of the PSC devices. If we used the same polymer:fullerene solutions (9 mg/mL), high temperature and high spinrate conditions would result in thin active layers (100-150 nm), which makes the comparison unfair. Therefore, we process the 14 mg/mL solution of PffBT4T-2OD:$PC_{61}PM$ at higher temperatures and higher spinrates to obtain similar thicknesses (~260 nm) to that of our optimized device. The device results are compared in Supplementary Table 4. Using the fast quenching conditions (high temperature and high rate), the crystallinity of the polymer is poor (evidenced by our XRD data, similar to the green line in Figure 2d) and there is also a significant decrease in the intensity and blue-shift of energy for the 0-0 transition peak (Figure 2e). As a result, the corresponding thick-film PSCs exhibit poor efficiency

(3-5%, Supplementary Table 4) due to, among other factors, significantly lower hole mobility of the film and poor polymer:fullerene morphology.

Supplementary Methods

General Information.

All reagents and chemicals were purchased from commercial sources and used without further purification unless stated otherwise. Tetrahydrofuran (THF) was freshly distilled before use from sodium using benzophenone as indicator. Pyridine was freshly distilled before use from potassium hydroxide. Anhydrous 1,2-dichlorobenzene and chlorobenzene were purchased from J&K Chemical and Sigma-Aldrich, respectively, and used as received. ^{1}H and ^{13}C NMR spectra were recorded on a Bruker AV-400 MHz NMR spectrometer. Chemical shifts are reported in parts per million (ppm, δ). ^{1}H NMR and ^{13}C NMR spectra were referenced to tetramethylsilane (0 ppm) for $CDCl_3$, or solvent residual peak (5.98 ppm, ^{1}H NMR only) for $C_2D_2Cl_4$ as internal standard. ^{19}F NMR spectra were referenced to 0.05% α,α,α-trifluorotoluene in $CDCl_3$ (-64 ppm) as external standard. Mass spectra were collected on a MALDI Micro MX mass spectrometer, or an API QSTAR XL System. Elemental analysis was performed by Midwest Microlab, LLC. Polymer molecular weights were determined on a Polymer Laboratories PL-GPC 220 using trichlorobenzene as eluent at 170 °C vs polystyrene standards.

Synthesis of Polymers.

4,7-dibromo-5,6-difluoro-2,1,3-benzothiadiazole (**S1**)[5], 3-(2-octyldodecyl)thiophene (**S2**)[6], 5,5'-bis(trimethylstannyl)-2,2'-bithiophene[7], 4,7-bis(5-bromo-4-(2-octyldodecyl)thiophen-2-yl)-2,1,3-benzothiadiazole[8] and 3,3'-difluoro-5,5'-bis(trimethylstannyl)-2,2'-bithiophene (**S5**)[9] were synthesized using literature reported procedures.

Synthesis of PffBT4T-2OD

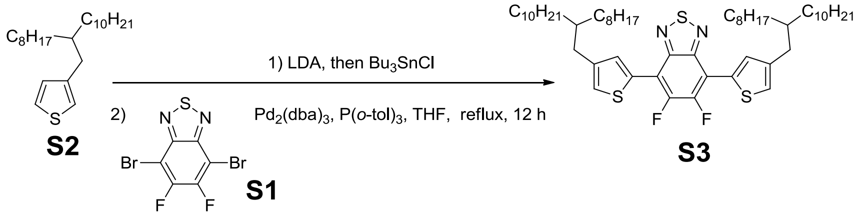

5,6-Difluoro-4,7-bis(4-(2-octyldodecyl)-2-thienyl)-2,1,3-benzothiadiazole (S3). A solution of 3-(2-octyldodecyl)thiophene (**S2**, 5.00 g, 13.7 mmol) in 50 mL THF was cooled to -78 °C under N_2. A solution of lithium diisopropylamide (2 M, 8.3 mL, 16.6 mmol) was added dropwise and the mixture was stirred at -78 °C for 1 h and then return to 0 °C and stirred for additional 1 h. Then the mixture was cooled to -78 °C and tri-*n*-butyltin chloride (6.50 g, 20 mmol) was added in one portion. The reaction mixture was return to r.t. and stirred overnight. A solution of KF in water was added and the organic phase was washed with water for three times, then dried with Na_2SO_4. The solvent was evaporated to get the crude product as yellow oil, which is directly used without further purification.

A mixture of 2-(tri-*n*-butylstannyl)-4-(2-octyldodecyl)thiophene (1.96 g, ~3 mmol), 4, 7-dibromo-5,6-difluoro-2,1,3-benzothiadiazole (**S1**, 330mg, 1 mmol), $Pd_2(dba)_3$ (11 mg, 0.02 mmol) and P(*o*-tol)$_3$ (24 mg, 0.08 mmol) in 10 mL THF was refluxed overnight under N_2. The reaction mixture was then cooled to r.t. and the solvent was evaporated. The residue was purified by flash column chromatography (eluent: *n*-hexane) to get the product as yellow solid (650 mg, 73%).

^{1}H NMR (400 MHz, $CDCl_3$) δ 8.11 (s, 2H), 7.19 (s, 2H), 2.66 (d, *J* = 6.7 Hz, 4H), 1.77 – 1.62 (m, 2H), 1.42 – 1.14 (m, 64H), 0.97 – 0.84 (m, 12H).

^{19}F NMR (376 MHz, $CDCl_3$) δ -128.27 (s, 2F).

^{13}C NMR (100 MHz, $CDCl_3$) δ 149.75 (dd, *J* = 257.9, 20.2 Hz), 148.94 (t, *J* = 4.1 Hz), 142.36, 132.81 (t, *J* = 3.8 Hz), 130.99, 124.83, 111.69 (dd, *J* = 9.2, 4.3 Hz), 38.97, 34.88, 33.34, 31.93, 30.05, 29.71, 29.67, 29.38, 26.66, 22.70, 14.12.

HRMS (MALDI+) Calcd for $C_{54}H_{86}F_2N_2S_3$ (M^+): 896.5921, Found: 896.5943.

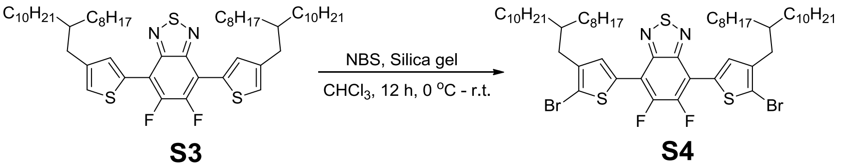

5,6-Difluoro-4,7-bis(5-bromo-4-(2-octyldodecyl)-2-thienyl)-2,1,3-benzothiadiazole (S4). *N*-Bromosuccinimide (540 mg, 3.00 mmol) was added to a mixture of **S3** (1.22 g, 1.36 mmol) and silica gel (20 mg) in 20 mL chloroform at 0 °C. The reaction mixture was warmed to r.t. and stirred overnight. After washed with water, the organic phase was dried with Na_2SO_4 and the solvent was evaporated. The residue was purified with flash column chromatography (eluent: *n*-hexane) to get the product as orange solid (1.42 g, 99%).

^{1}H NMR (400 MHz, $CDCl_3$) δ 7.94 (s, 2H), 2.60 (d, *J* = 7.1 Hz, 4H), 1.80 – 1.70 (m, 2H), 1.40 – 1.15 (m, 64H), 0.90 – 0.77 (m, 12H).

^{19}F NMR (376 MHz, $CDCl_3$) δ -128.10 (s, 2F).

^{13}C NMR (100 MHz, $CDCl_3$) δ 149.54 (dd, *J* = 258.9, 20.3 Hz), 148.26, 141.70, 132.25, 130.99, 124.83, 115.13 (t, *J* = 3.6 Hz), 110.85 (d, *J* = 8.6 Hz), 38.53, 34.08, 33.35, 31.94, 30.05, 29.73, 29.69, 29.67, 29.39, 26.56, 22.71, 14.13.

HRMS (MALDI+) Calcd for $C_{54}H_{84}Br_2F_2N_2S_3$ (M^+): 1052.4131, Found: 1052.4141.

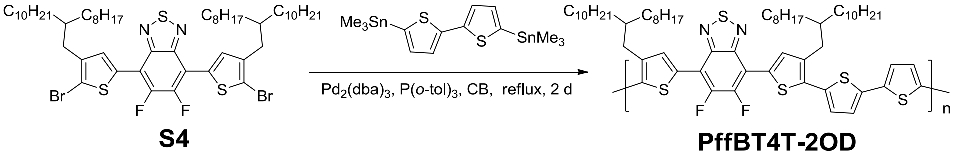

PffBT4T-2OD. The polymer can be synthesized by either microwave reaction or conventional reaction. To a mixture of monomer **S4** (100 mg, 0.095 mmol), 5,5'-bis(trimethylstannyl)-2,2'-bithiophene (46.7 mg, 0.095 mmol), $Pd_2(dba)_3$ (1.1 mg, 0.002 mmol) and P(*o*-tol)$_3$ (2.4 mg, 0.008 mmol) was added 1.6 mL of chlorobenzene in a glove box protected with N_2. The reaction mixture was then sealed and heated at 145 °C for 2 days (or at 160 °C for 30 min for microwave reaction). The mixture was

cooled to r.t. and 10 mL toluene was added before precipitated with methanol. The solid was collected by filtration, and loaded into an extraction thimble and washed successively with CH_2Cl_2 and $CHCl_3$. The polymer was finally collected from chlorobenzene. The chlorobenzene solution was then concentrated by evaporation, precipitated into methanol. The solid was collected by filtration and dried in vacuo to get the polymer as dark green solid (89 mg, 88%).

^{1}H NMR (400 MHz, $C_2D_2Cl_4$, 403 K). δ 8.20 (s, 2H), 7.26 (br, 4H), 2.95 (br, 4H), 1.93 (br, 2H), 1.56 – 1.21 (m, 64H), 1.00 – 0.83 (m, 12H).

Elem. Anal. Calcd for $C_{62}H_{88}F_2N_2S_5$: C, 70.27; H, 8.37; F, 3.59; N, 2.64; S, 15.13. Found: C, 70.33; H, 8.16; F, 3.70; N, 2.72; S, 14.91.

GPC Number-averaged molecular weight (M_n): 39-47 kDa; weight-averaged molecular weight (M_w): 72-94 kDa.

Synthesis of PBTff4T-2OD

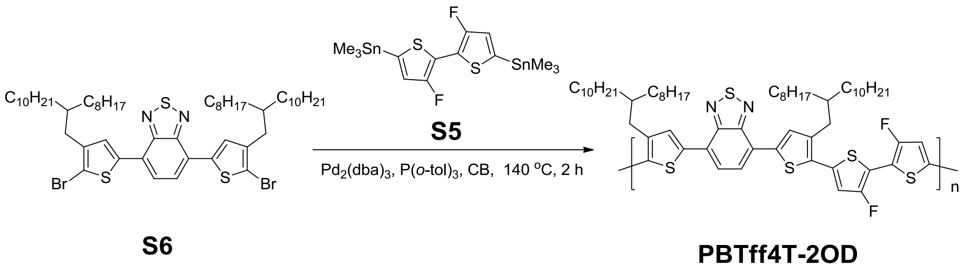

PBTff4T-2OD. To a mixture of monomer **S6** (20.4 mg, 0.020 mmol), **S5** (10.6 mg, 0.020 mmol), $Pd_2(dba)_3$ (0.5 mg, 0.0005 mmol) and P(*o*-tol)$_3$ (1.2 mg, 0.004 mmol) in a microwave vial equipped with a stirring bar was added 0.2 mL of chlorobenzene in a glove box protected with N_2. The reaction mixture was then sealed and heated to 140 °C for 2 hours using a microwave reactor. The mixture was cooled to r.t. and 5 mL of chlorobenzene was added before precipitated with methanol. The solid was collected by filtration, and loaded into an extraction thimble and washed with $CHCl_3$. The polymer was finally collected from chlorobenzene. The chlorobenzene solution was then concentrated by evaporation, precipitated into methanol. The solid was collected by filtration and dried in vacuo to get the polymer as dark green solid (15.0 mg, 71%).

^{1}H NMR (400 MHz, $C_2D_2Cl_4$, 393 K). δ 8.05 (s, 2H), 7.90 (s, 2H), 7.09 (s, 2H), 2.91 (d, J = 6.9 Hz, 4H), 1.92 (br, 2H), 1.56 – 1.22 (m, 64H), 1.00 – 0.87 (m, 12H).

Elem. Anal. Calcd for $C_{62}H_{88}F_2N_2S_5$: C, 70.27; H, 8.37; N, 2.64; S, 15.13. Found: C, 70.38; H, 8.36; N, 2.71; S, 14.82.

GPC M_n=28.2 kDa; M_w=52.9 kDa; PDI=1.88.

Synthesis of PNT4T-2OD

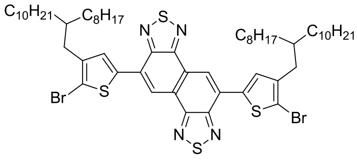

S7

4,9-Bis(5-bromo-4-(2-decyltetradecyl)thiophen-2-yl)naphtho[1,2-*c*:5,6*c'*]bis[1,2,5] thiadiazole (S7) was synthesized according to the literature procedure.[8]

^{1}H NMR (400 MHz, $CDCl_3$) δ 8.96 (s, 2H), 7.92 (s, 2H), 2.61 (d, J = 7.2 Hz, 4H), 1.80 (br, 2H), 1.45 – 1.12 (m, 64H), 0.91 – 0.79 (m, 12H).

^{13}C NMR (100 MHz, $CDCl_3$) δ 153.18, 151.99, 142.41, 137.90, 129.40, 125.77, 124.57, 121.40, 113.33, 38.63, 34.36, 33.42, 33.40, 31.94, 31.93, 30.10, 29.74, 29.72, 29.69, 29.39, 29.38, 26.59, 22.69, 14.12.

HRMS (MALDI+) Calcd for $C_{46}H_{68}Br_2F_2N_2S_3$: 1124.4102, Found: 1124.4117.

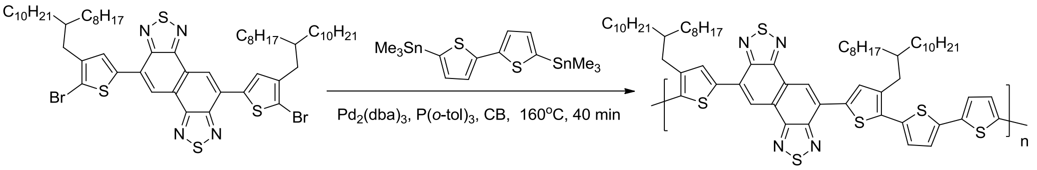

S7 **PNT4T-2OD**

PNT4T-2OD. To a mixture of monomer **S7** (27.4 mg, 0.024 mmol), 5,5'-bis(trimethylstannyl)-2,2'-bithiophene (12.0 mg, 0.024 mmol), $Pd_2(dba)_3$ (0.5 mg, 0.0005 mmol) and P(*o*-tol)$_3$ (1.2mg, 0.004mmol) in a microwave vial equipped with a stirring bar was added 0.2 mL of chlorobenzene in a glove box protected with N_2. The reaction mixture was then sealed and heated to 160 °C for 40 min using a microwave reactor. The mixture was cooled to r.t. and 5 mL of chlorobenzene was added before precipitated with methanol. The solid was collected by filtration, and loaded into an extraction thimble and washed with $CHCl_3$. The polymer was finally collected from chlorobenzene. The chlorobenzene solution was then concentrated by evaporation, precipitated into methanol. The solid was collected by filtration and dried in vacuo to get the polymer as dark green solid (15.8 mg, 56%).

^{1}H NMR (400 MHz, $C_2D_2Cl_4$, 408 K). δ 9.20 – 8.52 (m, 2H), 8.28 – 8.05 (m, 2H), 7.35 – 7.10 (m, 2H), 2.97 (br, 4H), 2.00 (br, 2H), 1.70 – 1.15 (m, 64H), 1.00 – 0.85 (m, 12H).

Elem. Anal. Calcd for $C_{66}H_{90}N_4S_6$: C, 70.04; H, 8.02; N, 4.95. Found: C, 70.22; H, 8.03; N, 4.96.

GPC M_n=30.2 kDa; M_w=49.9 kDa; PDI=1.65.

Synthesis of PffBT4T-1ON

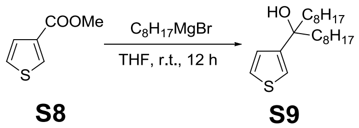

S8 **S9**

9-(Thiophen-3-yl)heptadecan-9-ol (S9). To a solution of octylmagnesium bromide in THF (14 mmol, 30 mL) was added methyl thiophene-3-carboxylate (**S8**, 500 mg, 3.5 mmol) in one portion at r.t., the reaction mixture was stirred at r.t. overnight before quenched with 1 M HCl (aq.). The organic phase was separated and dried with anhydrous Na_2SO_4. The solvent was removed and the crude product was purified by flash column chromatography (eluent: *n*-hexane: CH_2Cl_2 = 1:1) to get the product as colourless oil. (623 mg, 53 %).
^{1}H NMR (400 MHz, $CDCl_3$) δ 7.38 – 7.21 (m, 1H), 7.21 – 7.03 (m, 1H), 7.03 – 6.87 (m, 1H), 1.85 – 1.67 (m, 4H), 1.46 – 1.07 (m, 24H), 0.97 – 0.81 (m, 6H).
^{13}C NMR (100 MHz, $CDCl_3$) δ 148.91, 125.78, 125.43, 119.74, 42.49, 31.89, 31.63, 30.03, 29.55, 29.30, 23.56, 22.68, 14.13.
HRMS (CI+) Calcd for $C_{21}H_{38}OS$ (M^+): 338.2643, Found: 338.2653.

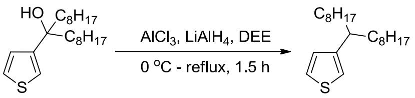

S9 **S10**

3-(Heptadecan-9-yl)thiophene (S10). To a suspension of $AlCl_3$ (160 mg, 1.2 mmol) in diethyl ether (0.5 mL) was added a solution of $LiAlH_4$ in THF (2 M, 0.6 mL) at 0 °C under N_2. **S9** (100 mg, 0.3 mmol) in diethyl ether (0.5 mL) was added to the solution and the reaction mixture was refluxed for 1.5 h before quenched with 1 M HCl (aq.). The organic phase was separated and dried with anhydrous Na_2SO_4. The solvent was removed and the crude product was purified by flash column chromatography (eluent: *n*-hexane) to get the product as colourless oil. (91 mg, 96%).
^{1}H NMR (400 MHz, $CDCl_3$) δ 7.31 – 7.25 (m, 1H), 6.95 (dd, J = 4.9, 1.1 Hz, 1H), 6.91 (dd, J = 2.9, 1.1 Hz, 1H), 2.74 – 2.62 (m, 1H), 1.71 – 1.47 (m, 4H), 1.26 (dd, J = 28.5, 13.7 Hz, 24H), 1.00 – 0.82 (m, 6H).
HRMS (CI+) Calcd for $C_{21}H_{38}S$ (M^+): 322.2694, Found: 322.2696.

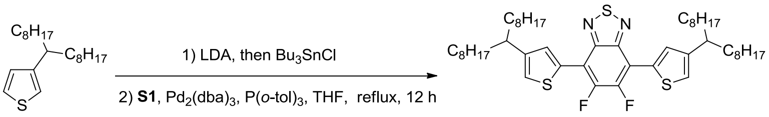

S10 **S11**

5,6-Difluoro-4,7-bis(4-(heptadecan-9-yl)-2-thienyl)-2,1,3-benzothiadiazole (S11). A solution of 3-(heptadecan-9-yl)thiophene (**S10**, 91.3 mg, 0.28 mmol) in 1 mL THF was cooled to -78 °C under N_2. A solution of lithium diisopropylamide (2 M, 0.2 mL, 0.4 mmol) was added dropwise and the mixture was stirred at -78 °C for 1 h and then return to 0 °C and stirred for additional 1 h. Then the mixture was cooled to -78 °C and tri-*n*-butyltin chloride (0.13 mL, 0.5 mmol) was added in one portion. The reaction mixture was return to r.t. and stirred overnight. A solution of KF in water was

added and the organic phase was washed with water for three times, then dried with Na_2SO_4. The solvent was evaporated to get the crude product as yellow oil, which is directly used without further purification.

A mixture of 2-(tri-*n*-butylstannyl)-4-(heptadecan-9-yl)thiophene (190 mg, ~0.28 mmol), 4,7-dibromo-5,6-difluoro-2,1,3-benzothiadiazole **S1** (33 mg, 0.1 mmol), $Pd_2(dba)_3$ (6 mg, 0.01 mmol) and P(*o*-tol)$_3$ (12 mg, 0.04 mmol) in 0.5 mL THF was refluxed overnight under N_2. The reaction mixture was then cooled to r.t. and the solvent was evaporated. The residue was purified by flash column chromatography (eluent: *n*-hexane) to get the product as yellow solid (16 mg, 20%).

^{1}H NMR (400 MHz, $CDCl_3$) δ 8.11 (s, 2H), 7.18 (s, 2H), 2.77 – 2.66 (m, 2H), 1.73 – 1.51 (m, 8H), 1.32 – 1.18 (m, 48H), 0.85 (t, *J* = 6.7 Hz, 12H).

^{19}F NMR (376 MHz, $CDCl_3$) δ -128.27 (s, 2F).

^{13}C NMR (100 MHz, $CDCl_3$) *δ* 149.11 (dd, *J* = 258.2, 20.7 Hz), 148.40 – 148.20 (m), 147.19, 130.52, 130.47 – 130.30 (m), 123.08, 111.10 (dd, *J* = 9.3, 4.4 Hz), 40.74, 35.95, 31.24, 29.09, 28.90, 28.67, 26.89, 22.02, 13.46.

HRMS (CI+) Calcd for $C_{48}H_{74}F_2N_2S_3$ (M^+): 812.4982, Found: 812.5003.

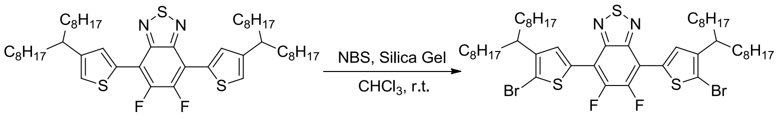

S11 S12

4,7-Bis(5-bromo-4-(heptadecan-9-yl)thiophen-2-yl)-5,6-difluorobenzothiadiazole (S12). *N*-Bromosuccinimide (7.9 mg, 0.044 mmol) was added to a mixture of **S11** (15 mg, 0.018 mmol) and a small amount of silica gel in 0.5 mL chloroform at 0 °C. The reaction mixture was warmed to r.t. and stirred overnight. After washed with water, the organic phase was dried with Na_2SO_4 and the solvent was evaporated. The residue was purified with flash column chromatography (eluent: *n*-hexane) to get the product as orange solid (16 mg, 92%).

^{1}H NMR (400 MHz, $CDCl_3$) *δ* 7.92 (s, 2H), 3.07 – 2.66 (m, 2H), 1.73 – 1.52 (m, 8H), 1.37 – 1.07 (m, 48H), 0.85 (t, *J* = 6.8 Hz, 12H).

^{19}F NMR (376 MHz, $CDCl_3$) *δ* -128.08 (s, 2F).

^{13}C NMR (100 MHz, $CDCl_3$) *δ* 149.09 (dd, *J* = 258.8, 20.1 Hz), 147.83 (d, *J* = 4.3 Hz), 145.60, 131.00, 128.87 (t, *J* = 4.4 Hz), 114.17, 110.53 (dd, *J* = 8.9, 3.8 Hz), 39.22, 35.55, 31.22, 29.01, 28.85, 28.65, 26.74, 22.01, 13.45.

HRMS (MALDI+) Calcd for $C_{48}H_{72}Br_2F_2N_2S_3$: 968.3192, Found: 968.3321.

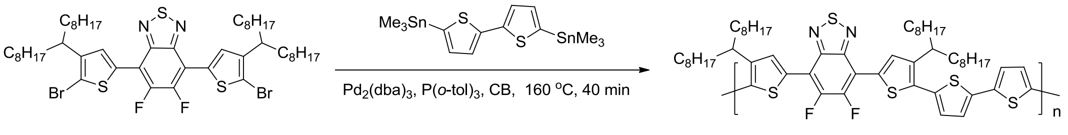

S12 PffBT4T-1ON

PffBT4T-1ON. To a mixture of monomer **S12** (14.8 mg, 0.015 mmol), 5,5'-bis(trimethylstannyl)-2,2'-bithiophene (7.5 mg, 0.015 mmol), $Pd_2(dba)_3$ (0.6 mg, 0.0007 mmol) and P(*o*-tol)$_3$ (1.2 mg, 0.004 mmol) in a microwave vial equipped with

a stirring bar was added 0.1 mL of chlorobenzene in a glove box protected with N_2. The reaction mixture was then sealed and heated to 160 °C for 40 min using a microwave reactor. The mixture was cooled to r.t. and 5 mL of chlorobenzene was added before precipitated with methanol. The solid was collected by filtration, and loaded into an extraction thimble and washed with ethyl acetate followed by $CHCl_3$. The polymer was finally collected from chlorobenzene. The chlorobenzene solution was then concentrated by evaporation, precipitated into methanol. The solid was collected by filtration and dried in vacuo to get the polymer as dark purple solid (4.8 mg, 32%).

^{1}H NMR (400 MHz, $C_2D_2Cl_4$, 333 K). δ 8.18 (s, 2H), 7.26 (br, 2H), 7.22 (br, 2H), 3.24 (br, 2H), 1.75 (br, 8H), 1.41 – 1.12 (m, 48H), 0.87 (t, J = 6.6 Hz, 12H).

GPC M_n=31.8 kDa; M_w=52.3 kDa; PDI=1.64.

Synthesis of PffBT4T-3OT

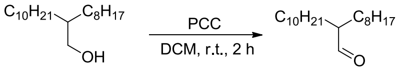

S13 **S14**

2-Octyldodecanal (S14). Pyridinium chlorochromate (43.3 g, 201 mmol) was suspended in CH_2Cl_2 (220 mL). **S13** (40.0 g, 134 mmol) is added dropwise. The mixture was stirred at r.t. for 2h, after which it was poured onto a silica gel pad and washed with CH_2Cl_2. The solvent was removed to afford the product as colourless oil without further purification (37.7 g, 95%).

^{1}H NMR (400 MHz, $CDCl_3$) δ 9.54 (d, J = 3.2 Hz, 1H), 2.26 – 2.15 (m, 1H), 1.67 – 1.13 (m, 32H), 0.87 (t, J = 6.8 Hz, 6H).

^{13}C NMR (100 MHz, $CDCl_3$) δ 206.13, 32.24, 32.18, 30.04, 29.93, 29.91, 29.77, 29.74, 29.66, 29.57, 29.26, 27.42, 23.02, 22.99, 14.44, 14.42.

HRMS (CI+) Calcd for $C_{20}H_{40}O_2$ ($M+O^+$): 312.3028, Found: 312.3028.

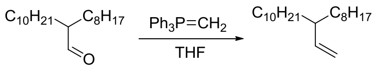

S14 **S15**

9-Vinylnonadecane (S15). A solution of methyltriphenylphosphonium bromide (2.80 g, 7.8 mmol) in 30 mL THF was cooled to -78 °C under N_2. *n*-Butyllithium (4.9 mL, 7.8 mmol) was added dropwise and the mixture was kept at -78 °C for 15 min and warmed to r.t. for another 10 min. the reaction mixture was then cooled to -78 °C again, 2-octyldodecanal (**S14**) (2.30 g, 7.8 mmol) was added dropwise and the mixture was stirred at -78 °C for 15 min before warmed to r.t. for another 1 h. The reaction mixture was then filtered through Celite, the filtrate was concentrated and purified by flash column chromatography (eluent: *n*-hexane) to get the product as colourless oil. (745 mg, 32%).

^{1}H NMR (400 MHz, $CDCl_3$) δ 5.59 – 5.44 (m, 1H), 4.99 – 4.86 (m, 2H), 2.06 – 1.81 (m, 1H), 1.46 – 1.02 (m, 32H), 0.96 – 0.78 (m, 6H).

^{13}C NMR (100 MHz, $CDCl_3$) δ 143.78, 113.73, 44.11, 35.06, 31.95, 29.82, 29.71, 29.67, 29.64, 29.37, 27.20, 22.71, 14.13.
HRMS (CI+) Calcd for $C_{21}H_{42}$ (M^+): 294.3287, Found: 294.3283.

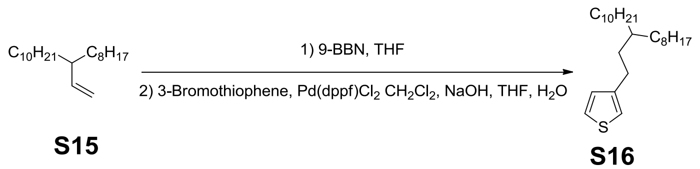

3-(3-Octyltridecanyl)thiophene (S16). To a solution of 9-borabicyclo[3.3.1]nonane (6.2 mL, 0.5 M in THF) was added 9-vinylnonadecane (**S15**) (653 mg, 2.22 mmol) under N_2, and the mixture was stirred at r.t. overnight. $Pd(dppf)Cl_2 \cdot CH_2Cl_2$ (125 mg , 0.15 mmol) in 40 mL THF together with an aqueous NaOH solution (1.5 M, 2mL) was added to the mixture. Then the mixture was refluxed for 15 h under N_2. After cooled to r.t., the organic phase was separated and the aqueous solution was extracted with diethyl ether. The combined organic phase was washed with water and then brine before dried with anhydrous Na_2SO_4. The solvent was evaporated under reduced pressure. The residue was purified by flash column chromatography (eluent: *n*-hexane) to get pure product as colourless oil. (330 mg, 39%).
^{1}H NMR (400 MHz, $CDCl_3$) δ 7.23 (dd, *J* = 4.9, 2.9 Hz, 1H), 7.00 – 6.86 (m, 2H), 2.68 – 2.55 (m, 2H), 1.62 – 1.52 (m, 2H) 1.47 – 1.04 (m, 33H), 0.92 – 0.84 (m, 6H).
^{13}C NMR (100 MHz, $CDCl_3$) δ 143.58, 128.30, 125.05, 119.57, 37.12, 34.51, 33.52, 31.94, 30.13, 29.71, 29.67, 29.37, 27.53, 26.63, 22.70, 14.13.
HRMS (CI+) Calcd for $C_{25}H_{46}S$ (M^+): 378.3320, Found: 378.3318.

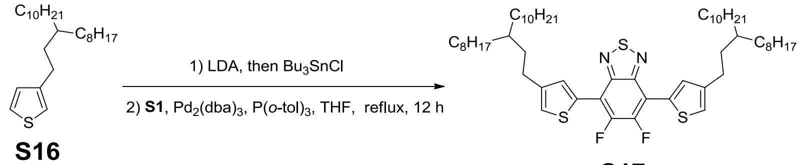

5,6-Difluoro-4,7-bis(4-(3-octyltridecanyl)-2-thienyl)-2,1,3-benzothiadiazole (S17). A solution of 3-(3-octyltridecanyl)thiophene (**S16**) (378 mg, 1.0 mmol) in 4 mL THF was cooled to -78 °C under N_2. A solution of lithium diisopropylamide (2 M, 0.6 mL, 1.2 mmol) was added dropwise and the mixture was stirred at -78 °C for 1 h and then return to 0 °C and stirred for additional 1 h. Then the mixture was cooled to -78 °C and tri-*n*-butyltin chloride (0.4 mL, 1.5 mmol) was added in one portion. The reaction mixture was return to r.t. and stirred overnight. A solution of KF in water was added and the organic phase was washed with water for three times, then dried with Na_2SO_4. The solvent was evaporated to get the crude product as yellow oil, which is directly used without further purification.
A mixture of 2-(tri-*n*-butylstannyl)-4-(3-octyltridecanyl) thiophene (700 mg, ~1 mmol), 4,7-dibromo-5,6-difluoro-2,1,3-benzothiadiazole (**S1**, 131.2 mg, 0.41 mmol), $Pd_2(dba)_3$ (11 mg, 0.02 mmol) and $P(o\text{-tol})_3$ (24 mg, 0.08 mmol) in 1 mL THF was refluxed overnight under N_2. The reaction mixture was then cooled to r.t. and the

solvent was evaporated. The residue was purified by flash column chromatography (eluent: *n*-hexane) to get the product as yellow solid (101 mg, 27%).

^{1}H NMR (400 MHz, $CDCl_3$) δ 8.12 (d, *J* = 1.3 Hz, 2H), 7.21 (s, 2H), 2.82 – 2.64 (m, 4H), 1.74 – 1.60 (m, 4H), 1.48 – 1.18 (m, 66H), 0.93 – 0.86 (m, 12H).

^{19}F NMR (376 MHz, $CDCl_3$) δ -128.23 (s, 2F).

HRMS (MALDI+) Calcd for $C_{56}H_{90}F_2N_2S_3$ (M^+): 924.6234, Found: 924.6244.

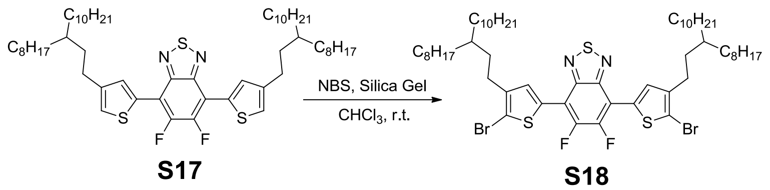

5,6-Difluoro-4,7-bis(5-bromo-4-(3-octyltridecanyl)-2-thienyl)-2,1,3-benzothiadiazole (S18). *N*-Bromosuccinimide (46.6 mg, 0.26 mmol) was added to a mixture of **S17** (101 mg, 0.11 mmol) and a small amount of silica gel in 3 mL chloroform at 0 °C. The reaction mixture was warmed to r.t. and stirred overnight. After washed with water, the organic phase was dried with Na_2SO_4 and the solvent was evaporated. The residue was purified with flash column chromatography (eluent: *n*-hexane) to get the product as orange solid (90 mg, 76%).

^{1}H NMR (400 MHz, $CDCl_3$) δ 7.94 (s, 2H), 2.67 – 2.59 (m, 4H), 1.65 – 1.57 (m, 4H), 1.43-1.14 (m, 66H), 0.87 (t, *J* = 6.8 Hz, 12H).

^{19}F NMR (376 MHz, $CDCl_3$) δ -128.06 (s, 2F).

^{13}C NMR (100 MHz, $CDCl_3$) δ 149.67 (dd, *J* = 258.8, 20.3 Hz), 148.40 (d, *J* = 3.7 Hz), 142.92, 131.74, 131.29, 114.34, 111.03 (dd, *J* = 8.9, 4.4 Hz), 37.10, 33.60, 33.48, 31.95, 30.13, 29.76, 29.74, 29.72, 29.69, 29.40, 26.92, 26.66, 22.71, 14.14.

HRMS (MALDI+) Calcd for $C_{56}H_{88}Br_2F_2N_2S_3$: 1080.4444, Found: 1080.4478.

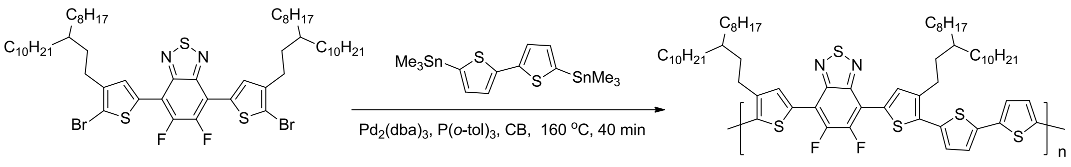

PffBT4T-3OT. To a mixture of monomer **S18** (20.9 mg, 0.019 mmol), 5,5'-bis(trimethylstannyl)-2,2'-bithiophene (9.7 mg, 0.020 mmol), $Pd_2(dba)_3$ (0.6 mg, 0.0007 mmol) and P(*o*-tol)$_3$ (1.2 mg, 0.004 mmol) in a microwave vial equipped with a stirring bar was added 0.1 mL of chlorobenzene in a glove box protected with N_2. The reaction mixture was then sealed and heated to 160 °C for 40 min using a microwave reactor. The mixture was cooled to r.t. and 5 mL of chlorobenzene was added before precipitated with methanol. The solid was collected by filtration, and loaded into an extraction thimble and washed with $CHCl_3$. The polymer was finally collected from chlorobenzene. The chlorobenzene solution was then concentrated by evaporation, precipitated into methanol. The solid was collected by filtration and dried in vacuo to get the polymer as dark green solid (15.2 mg, 72%).

^{1}H NMR (400 MHz, $C_2D_2Cl_4$, 408 K). δ 8.21 (s, 2H), 7.28 (s, 4H), 3.05 – 2.92 (m, 4H), 1.87 – 1.77 (br, 4H), 1.61 – 1.20 (m, 66H), 1.01 – 0.88 (m, 12H).
GPC M_n=21.1 kDa; M_w=36.3 kDa; PDI=1.72.

Synthesis of fullerenes.

$PC_{61}PM$[10], $PC_{61}BM$[11], $TC_{61}BM$[12], PFP[13], TFP[14], NCMA[15], NCMM[16], ICMA[17], $PC_{71}BM$[18] were synthesized according to the literature procedures.

Synthesis of hydrazones.

Methyl 2-oxo-2-phenylacetate-*p*-tosylhydrazone[19] and methyl 5-oxo-5-(2-thienyl)pentanoate-*p*-tosylhydrazone[12] were prepared according to the literature procedures.

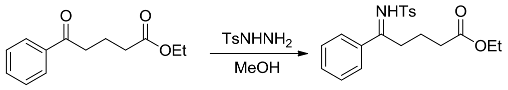

S19 **S20**

Ethyl 5-oxo-5-phenylpentanoate-*p*-tosylhydrazone (S20). A mixture of ethyl 5-oxo-5-phenylpentanoate (**S19**) (466 mg, 2.12 mmol), *p*-toluene-sulfonyl hydrazide (394 mg, 2.12 mmol), and MeOH (7 mL) was refluxed overnight. The mixture was cooled to -20 °C. The precipitate was collected by filtration and washed with MeOH. The product was obtained as white solid without further purification (460 mg, 56%).
^{1}H NMR (400 MHz, $CDCl_3$) δ 9.30 (s, 1H), 7.91 (d, J = 8.2 Hz, 2H), 7.65 (dd, J = 6.6, 3.0 Hz, 2H), 7.33 (m, 5H), 4.27 (q, J = 7.1 Hz, 2H), 2.68 – 2.59 (m, 2H), 2.40 (s, 3H), 2.34 – 2.27 (m, 2H), 1.73 – 1.62 (m, 2H), 1.31 (t, J = 7.1 Hz, 3H).
^{13}C NMR (100 MHz, $CDCl_3$) δ 173.84, 152.95, 143.06, 135.54, 135.43, 128.84, 127.80, 127.31, 125.57, 60.79, 31.63, 25.22, 20.96, 20.34, 13.59.
HRMS (MALDI+) Calcd for $C_{20}H_{25}N_2O_4S$ ($M+H^+$): 389.1535, Found: 389.1536.

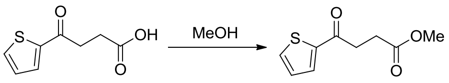

S21 **S22**

Methyl 4-oxo-4-(2-thienyl)butanoate (S22). A solution of 4-oxo-4-(2-thienyl)butyric acid (**S21**) (1.84 g, 10 mmol) in MeOH (20 mL) with a catalytic amount of hydrochloric acid was refluxed overnight. The mixture was cooled to r.t., diluted with H_2O and extracted with CH_2Cl_2. The organic phase was washed with H_2O, brine, dried with Na_2SO_4, filtered and concentrated in vacuo. The residue was purified by flash column chromatography (eluent: *n*-hexane/ethyl acetate = 5/1). The product was obtained as transparent oil (1.81 g, 91%).

^{1}H NMR (400 MHz, $CDCl_3$) δ 7.77 (d, J = 3.8 Hz, 1H), 7.65 (d, J = 4.9 Hz, 1H), 7.19 – 7.08 (m, 1H), 3.70 (s, 3H), 3.27 (t, J = 6.7 Hz, 2H), 2.77 (t, J = 6.7 Hz, 2H).
^{13}C NMR (100 MHz, $CDCl_3$) δ 190.98, 173.17, 143.62, 133.68, 132.00, 128.13, 51.89, 33.94, 27.99.
HRMS (CI+) Calcd for $C_9H_{10}O_3S$ (M^+): 198.0351, Found: 198.0349.

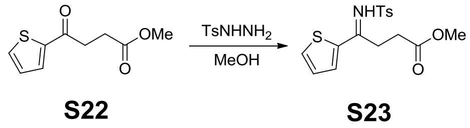

Methyl 4-oxo-4-(2-thienyl)butanoate-*p*-tosylhydrazone (S23). A mixture of **S22** (595 mg, 3 mmol), *p*-toluene-sulfonyl hydrazide (559 mg, 3 mmol), and MeOH (3 mL) was refluxed overnight. The mixture was cooled to -20 °C. The precipitate was collected by filtration and washed with MeOH. The product was obtained as white solid without further purification (800 mg, 73%).
^{1}H NMR (400 MHz, $CDCl_3$) δ 9.71 (s, 1H), 7.93 (d, J = 8.2 Hz, 2H), 7.33 – 7.27 (m, 3H), 7.09 (d, J = 3.7 Hz, 1H), 6.99 – 6.94 (m, 1H), 3.59 (s, 3H), 2.93 – 2.82 (m, 2H), 2.75 – 2.65 (m, 2H), 2.40 (s, 3H).
^{13}C NMR (100 MHz, $CDCl_3$) δ 174.09, 149.95, 143.06, 140.88, 134.83, 128.56, 127.82, 127.64, 126.51, 125.39, 51.90, 30.75, 21.51, 20.97.
HRMS (MALDI+) Calcd for $C_{16}H_{19}N_2O_4S_2$ ($M+H^+$): 367.0786, Found: 367.0760.

Synthesis of fullerenes.

General Procedure for the Preparation of Fullerene Derivatives.
Hydrazone (0.208 mmol) and MeONa (12.0 mg, 0.222 mmol) were suspended in dry pyridine (4 mL) under N_2 and the mixture was stirred at room temperature for ~30 min. Then a solution of C_{60} (100 mg, 0.139 mmol) in *o*-DCB (14 mL) was added. The reaction mixture was heated to 180 °C overnight before cooled to r.t. The solvent was evaporated under reduced pressure. The residue was purified by flash column chromatography twice (eluent: CS_2/CH_2Cl_2 = 2/1 for the first column, toluene for the second one). The obtained solid was dissolved in a minimal amount of hot $CHCl_3$ and subsequently precipitated with MeOH. The precipitate was collected by filtration and dried in vacuo at ~80 °C to give the product.

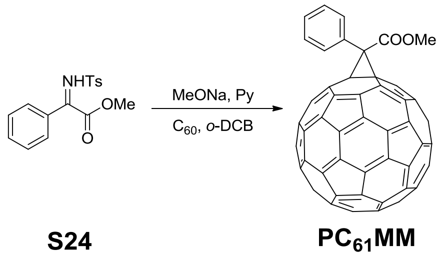

[6,6]-Phenyl-C61-methanoic acid methyl ester (PC$_{61}$MM) was prepared as brown powder from methyl 2-oxo-2-phenylacetate-*p*-tosylhydrazone (**S24**) (60.8 mg, 0.183

mmol) and 10.4 mg MeONa (0.192 mmol) according to the General Procedure (9.1 mg, 8%).

^{1}H NMR (400 MHz, $CDCl_3$) *δ* 8.12 (dd, *J* = 8.0, 1.5 Hz, 2H), 7.63 – 7.49 (m, 3H), 3.98 (s, 3H).

^{13}C NMR (100 MHz, $CDCl_3$) *δ* 167.24, 147.41, 146.27, 145.51, 145.22, 145.18, 145.14, 144.73, 144.70, 144.52, 144.47, 144.37, 143.91, 143.72, 143.12, 143.04, 142.99, 142.92, 142.40, 142.19, 142.14, 142.07, 140.97, 138.29, 137.84, 132.39, 132.35, 129.40, 128.74, 75.54, 55.84, 53.86.

HRMS (MALDI+) Calcd for $C_{69}H_8O_2$ (M^+): 868.0524, Found: 868.0542.

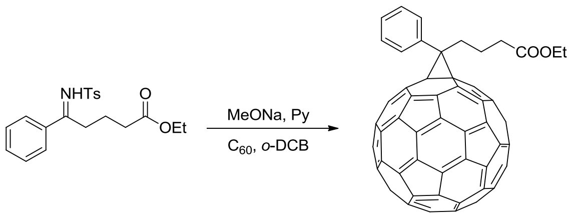

S20 PC61BE

[6,6]-Phenyl-C61-butyric acid ethyl ester (PC$_{61}$BE) was prepared as brown powder from **S20** (120 mg, 0.309 mmol), 18.0 mg MeONa (0.333 mmol) and 200 mg C_{60} (0.238 mmol) according to the General Procedure (97.9 mg, 44%).

^{1}H NMR (400 MHz, $CDCl_3$) *δ* 7.93 (d, *J* = 7.2 Hz, 2H), 7.55 (t, *J* = 7.5 Hz, 2H), 7.47 (t, *J* = 7.3 Hz, 1H), 4.14 (q, *J* = 7.1 Hz, 2H), 2.97 – 2.85 (m, 2H), 2.51 (t, *J* = 7.5 Hz, 2H), 2.25 – 2.10 (m, 2H), 1.25 (t, *J* = 7.1 Hz, 3H).

^{13}C NMR (100 MHz, $CDCl_3$) *δ* 173.09, 148.84, 147.83, 145.87, 145.20, 145.16, 145.09, 145.04, 144.80, 144.69, 144.67, 144.51, 144.43, 144.01, 143.77, 143.04, 143.00, 142.94, 142.24, 142.18, 142.14, 142.12, 140.99, 140.75, 138.05, 137.57, 136.76, 132.12, 128.44, 128.24, 79.89, 60.55, 51.89, 34.15, 33.68, 22.38, 14.27.

HRMS (MALDI+) Calcd for $C_{73}H_{16}O_2$ (M^+): 924.1150, Found: 924.1187.

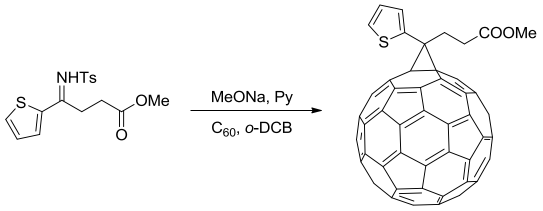

S23 TC61PM

[6,6]-(2-Thienyl)-C61-propanoic acid methyl ester (TC$_{61}$PM) was prepared as brown powder from **S23** (76.3 mg, 0.208 mmol) according to the General Procedure (45.8 mg, 37%).

^{1}H NMR (400 MHz, $CDCl_3$) *δ* 7.51 – 7.47 (m, 2H), 7.14 (dd, *J* = 5.1, 3.6 Hz, 1H), 3.72 (s, 3H), 3.30 – 3.23 (m, 2H), 2.97 – 2.88 (m, 2H).

^{13}C NMR (100 MHz, $CDCl_3$) *δ* 172.82, 148.11, 147.08, 145.62, 145.27, 145.24, 145.16, 145.01, 144.84, 144.69, 144.64, 144.55, 144.22, 143.81, 143.07, 143.04,

142.97, 142.21, 142.18, 142.14, 141.01, 140.78, 138.55, 138.34, 138.21, 132.31, 126.58, 126.25, 79.63, 51.97, 44.84, 31.79, 30.05.
HRMS (MALDI+) Calcd for $C_{69}H_{10}O_2S$ (M^+): 902.0402, Found: 902.0445.

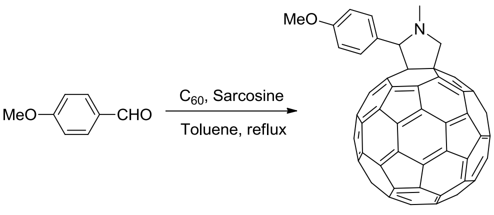

S25 **MOPFP**

***N*-Methyl-2-(4-methoxyphenyl) fulleropyrrolidine (MOPFP).** A solution of C_{60} (100 mg, 0.139 mmol), sarcosine (124 mg, 1.39 mmol), and *p*-anisaldehyde (**S25**) (94.4 mg, 0.694 mmol) in toluene (100 mL) was refluxed under N_2 for ~8h. The mixture was cooled to r.t. and the solvent was evaporated under reduced pressure. The residue was purified by flash column chromatography twice (eluent: CS_2/CH_2Cl_2 = 4/1 for the first column, toluene/*n*-hexane = 2:1 for the second one). The obtained solid was dissolved in a minimal amount of CS_2 and subsequently precipitated with *n*-hexane. The precipitate was collected by filtration and dried in vacuo at ~80 °C to give the product (28.9 mg, 24%).
^{1}H NMR (400 MHz, $CDCl_3$) δ 7.71 (br, 2H), 6.96 (d, J = 8.8 Hz, 2H), 4.98 (d, J = 9.4 Hz, 1H), 4.89 (s, 1H), 4.24 (d, J = 9.4 Hz, 1H), 3.82 (s, 3H), 2.79 (s, 3H).
^{13}C NMR (100 MHz, $CDCl_3/CS_2$) δ 159.02, 155.71, 153.39, 152.99, 152.89, 146.70, 146.17, 145.90, 145.73, 145.54, 145.35, 145.18, 145.03, 144.92, 144.84, 144.80, 144.67, 144.56, 144.08, 143.81, 142.57, 142.44, 142.11, 142.00, 141.68, 141.52, 141.46, 141.38, 141.27, 141.11, 140.99, 139.59, 139.41, 139.03, 136.23, 136.02, 135.20, 129.79, 128.18, 82.56, 69.45, 68.28, 54.33, 39.44.
HRMS (MALDI+) Calcd for $C_{70}H_{14}NO$ ($M+H^+$): 884.1075, Found: 884.1119.

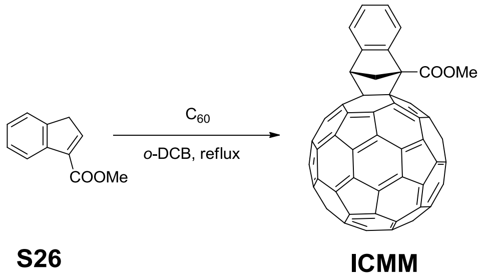

S26 **ICMM**

ICMA-methanoic acid methyl ester (ICMM). A solution of methyl 1*H*-indene-3-carboxylate (**S26**) (121 mg, 0.69 mmol) and C_{60} (250 mg, 0.35 mmol) in *o*-DCB (10 mL) was refluxed overnight. The reaction mixture was then cooled to r.t. and precipitated with methanol. The solid was collected by filtration and purified with flash column chromatography twice (eluent: toluene). The obtained solid was dissolved in a minimal amount of toluene and subsequently precipitated with MeOH. The precipitate was collected by filtration and dried in vacuo at ~80 °C to give the product as black powder (83 mg, 27%).

^{1}H NMR (400 MHz, $CDCl_3$) δ 7.93 – 7.87 (m, 1H), 7.65 (dd, J = 5.6, 2.8 Hz, 1H), 7.55 – 7.48 (m, 2H), 4.96 (s, 1H), 4.10 (dd, J = 10.5, 1.4 Hz, 1H), 3.99 (s, 3H), 3.18 (dd, J = 10.5, 1.7 Hz, 1H).
^{13}C NMR (100 MHz, $CDCl_3$) δ 170.11, 155.03, 153.69, 152.38, 152.10, 146.66, 145.75, 145.62, 145.48, 144.90, 144.77, 144.68, 144.59, 144.41, 143.87, 142.61, 142.39, 142.08, 141.92, 141.61, 141.50, 141.31, 141.21, 141.04, 139.66, 139.18, 139.07, 138.42, 137.20, 136.92, 136.65, 127.56, 127.07, 123.59, 123.25, 75.65, 68.83, 55.62, 51.98, 47.37.
HRMS (MALDI+) Calcd for $C_{71}H_{10}O_2$ (M^+): 894.0681, Found: 894.0673.

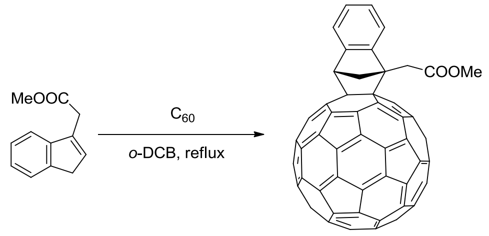

S27 **ICEM**

ICMA-ethanoic acid methyl ester (ICEM). A solution of methyl 1*H*-indene-3-acetate (**S27**) (70 mg, 0.372 mmol) and C_{60} (120 mg, 0.167 mmol) in *o*-DCB (12 mL) was refluxed overnight. The reaction mixture was then cooled to r.t. and precipitated with methanol. The solid was collected by filtration and purified with flash column chromatography twice (eluent: toluene). The obtained solid was dissolved in a minimal amount of $CHCl_3$ and subsequently precipitated with MeOH. The precipitate was collected by filtration and dried in vacuo at ~80 °C to give the product as brown solid (44 mg, 29%).
^{1}H NMR (400 MHz, $CDCl_3$) δ 7.57 – 7.51 (m, 1H), 7.51 – 7.45 (m, 1H), 7.41 – 7.34 (m, 2H), 4.89 (s, 1H), 3.98 (d, J = 15.1 Hz, 1H), 3.76 (dd, J = 10.3, 1.4 Hz, 1H), 3.73 – 3.65 (m, 4H), 3.25 (dd, J = 10.3, 1.7 Hz, 1H).
^{13}C NMR (100 MHz, $CDCl_3$) δ 170.97, 156.15, 154.50, 153.28, 152.62, 146.65, 146.58, 145.75, 145.68, 145.59, 145.51, 145.48, 145.43, 145.36, 145.06, 144.99, 144.89, 144.82, 144.79, 144.78, 144.55, 144.50, 144.33, 144.07, 143.89, 143.81, 143.66, 142.47, 142.21, 142.11, 142.05, 141.94, 141.91, 141.59, 141.50, 141.42, 141.40, 141.38, 141.29, 141.23, 141.16, 141.12, 139.61, 139.13, 138.92, 138.84, 137.29, 137.03, 136.58, 126.96, 126.68, 123.49, 121.97, 77.93, 61.58, 56.10, 51.36, 48.85, 35.72.
HRMS (MALDI+) Calcd for $C_{72}H_{13}O_2$ ($M+H^+$): 909.0916, Found: 909.0861.

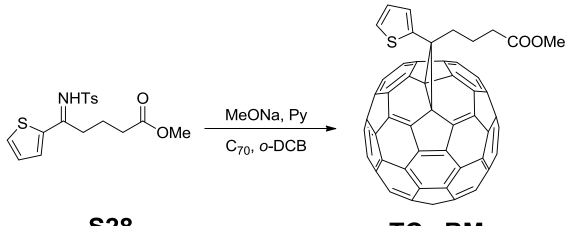

S28 **$TC_{71}BM$**

[6,6]-(2-Thienyl)-C71-butyric acid methyl ester ($TC_{71}BM$). Methyl 5-oxo-5-(2-thienyl)pentanoate-*p*-tosylhydrazone (**S28**) (70.6 mg, 0.186 mmol) and MeONa (10.8 mg, 0.200 mmol) were suspended in dry pyridine (4 mL) under N_2 and the mixture was stirred at room temperature for ~30 min. Then a solution of C_{70} (120 mg, 0.143 mmol) in *o*-DCB (18 mL) was added. The reaction mixture was heated to 180 °C overnight before cooled to r.t. The solvent was evaporated under reduced pressure. The residue was purified by flash column chromatography twice (eluent: CS_2/CH_2Cl_2 = 2/1 for the first column, toluene for the second one). The obtained solid was dissolved in a minimal amount of hot $CHCl_3$ and subsequently precipitated with MeOH. The precipitate was collected by filtration and dried in vacuo at ~80 °C to give the product as black powder (66.8 mg, 45%).

^{1}H NMR (400 MHz, $CDCl_3$) δ 7.48 (d, *J* = 3.4 Hz, 1H), 7.42 (d, *J* = 4.7 Hz, 1H), 7.09 (dd, *J* = 5.0, 3.7 Hz, 1H), 3.76 (s, minor isomer), 3.70 (s, 3H), 3.53 (s, minor isomer), 2.61 – 2.39 (m, 4H), 2.29 – 1.96 (m, 2H + minor isomers), 1.88 – 1.79 (m, minor isomers).

^{13}C NMR (100 MHz, $CDCl_3$) δ 173.44, 151.46, 150.90, 149.24, 148.51, 144.22, 143.62, 141.87, 140.32, 139.94, 137.64, 132.81, 131.57, 130.82, 126.16, 126.06, 72.43, 70.49, 51.75, 34.50, 33.61, 29.92, 21.83.

HRMS (MALDI+) Calcd for $C_{80}H_{12}O_2S$ (M^+): 1036.0558, Found: 1036.0575.

Supplementary References

1 Smilgies, D. M. Scherrer grain-size analysis adapted to grazing-incidence scattering with area detectors. *J. Appl. Crystallogr.* **42**, 1030-1034 (2009).

2 Wang, Y. *et al.* Substituent Effects on Physical and Photovoltaic Properties of 5,6-Difluorobenzo[c][1,2,5]thiadiazole-Based D-A Polymers: Toward a Donor Design for High Performance Polymer Solar Cells. *Macromolecules* **46**, 9587-9592 (2013).

3 Jheng, J. F. *et al.* Influences of the Non-Covalent Interaction Strength on Reaching High Solid-State Order and Device Performance of a Low Bandgap Polymer with Axisymmetrical Structural Units. *Adv. Mater.* **25**, 2445-2451 (2013).

4 Chen, Z. *et al.* Low Band-Gap Conjugated Polymers with Strong Interchain Aggregation and Very High Hole Mobility Towards Highly Efficient Thick-Film Polymer Solar Cells. *Adv. Mater.* **26**, 2586-2591 (2014).

5 Dou, L. *et al.* Synthesis of 5H-Dithieno[3,2-b:2′,3′-d]pyran as an Electron-Rich Building Block for Donor–Acceptor Type Low-Bandgap Polymers. *Macromolecules* **46**, 3384-3390 (2013).

6 Ong, K.-H. *et al.* Design and synthesis of benzothiadiazole–oligothiophene polymers for organic solar cell applications. *Polymer Chemistry* **4**, 1863 (2013).

7 Ha, J. S., Kim, K. H. & Choi, D. H. 2,5-Bis(2-octyldodecyl)pyrrolo[3,4-c]pyrrole-1,4-(2H,5H)-dione-Based Donor-Acceptor Alternating Copolymer Bearing 5,5 '-Di (thiophen-2-yl)-2,2 '-biselenophene Exhibiting 1.5 cm(2).V-1.s(-1) Hole Mobility in Thin-Film Transistors. *J. Am. Chem. Soc.* **133**, 10364-10367 (2011).

8 Osaka, I. *et al.* Synthesis, characterization, and transistor and solar cell applications of a naphthobisthiadiazole-based semiconducting polymer. *J. Am. Chem. Soc.* **134**, 3498-3507 (2012).

9 Jo, J. W. *et al.* Fluorination of Polythiophene Derivatives for High Performance Organic Photovoltaics. *Chem. Mater.* **26**, 4214-4220 (2014).

10 Mayorova, J. Y. *et al.* Synthesis and investigation of fullerene-based acceptor materials. *Mendeleev Commun.* **17**, 175-177 (2007).

11 Hummelen, J. C. *et al.* Preparation and Characterization of Fulleroid and Methanofullerene Derivatives. *J. Org. Chem.* **60**, 532-538 (1995).

12 Zhao, H. *et al.* Alkyl substituted [6,6]-thienyl-C61-butyric acid methyl esters: easily accessible acceptor materials for bulk-heterojunction polymer solar cells. *J. Mater. Chem.* **20**, 3092 (2010).

13 Zhang, X. *et al.* Flowerlike supramolecular architectures assembled from C60 equipped with a pyridine substituent. *Chem. Commun.* **46**, 8752-8754 (2010).

14 Chen, C., Li, X. & Yang, S. One-pot synthesis of new thio-derivatives of C60 with the unexpected formation of a thiazolidine-fulleropyrrolidine. *New J. Chem.* **34**, 331-336 (2010).

15 Kim, K.-H. *et al.* Facile Synthesis of o-Xylenyl Fullerene Multiadducts for High Open Circuit Voltage and Efficient Polymer Solar Cells. *Chem. Mater.* **23**, 5090-5095 (2011).

16 Backer, S. A., Sivula, K., Kavulak, D. F. & Fréchet, J. M. J. High Efficiency Organic Photovoltaics Incorporating a New Family of Soluble Fullerene Derivatives. *Chem. Mater.* **19**, 2927-2929 (2007).

17 Kang, H. *et al.* Controlling number of indene solubilizing groups in multiadduct fullerenes for tuning optoelectronic properties and open-circuit voltage in organic solar cells. *ACS Appl. Mater. Interfaces* **4**, 110-116 (2012).

18 Wienk, M. M. *et al.* Efficient methano[70]fullerene/MDMO-PPV bulk heterojunction photovoltaic cells. *Angew. Chem. Int. Ed.* **42**, 3371-3375 (2003).

19 Li, P., Zhao, J., Wu, C., Larock, R. C. & Shi, F. Synthesis of 3-Substituted Indazoles from Arynes and N-Tosylhydrazones. *Org. Lett.* **13**, 3340-3343 (2011).
